# Deoxyfluorination of acyl fluorides to trifluoromethyl compounds by FLUOLEAD^®^/Olah’s reagent under solvent-free conditions

**DOI:** 10.3762/bjoc.16.254

**Published:** 2020-12-14

**Authors:** Yumeng Liang, Akihito Taya, Zhengyu Zhao, Norimichi Saito, Norio Shibata

**Affiliations:** 1 Department of Nanopharmaceutical Sciences, Nagoya Institute of Technology, Gokiso, Showa-ku, Nagoya 466-5888, Japan; 2 Department of Life Science and Applied Chemistry, Nagoya Institute of Technology, Gokiso, Showa-ku, Nagoya 466-5888, Japan; 3 Pharmaceutical Division, Ube Industries, Ltd., Seavans North Bldg, 1-2-1 Shibaura, Minato-ku, Tokyo 105-8449, Japan; 4 Institute of Advanced Fluorine-Containing Materials, Zhejiang Normal University, 688 Yingbin Avenue, 321004 Jinhua, China

**Keywords:** acyl fluorides, deoxyfluorination, fluorine, solvent-free, trifluoromethyl group

## Abstract

A new protocol enabling the formation of trifluoromethyl compounds from acyl fluorides has been developed. The combination of FLUOLEAD^®^ and Olah’s reagent in solvent-free conditions at 70 °C initiated the significant deoxyfluorination of the acyl fluorides and resulted in the corresponding trifluoromethyl products with high yields (up to 99%). This strategy showed a great tolerance for various acyl fluorides containing aryloyl, (heteroaryl)oyl, or aliphatic acyl moieties, providing good to excellent yields of the trifluoromethyl products. Synthetic drug-like molecules were also transformed into the corresponding trifluoromethyl compounds under the same reaction conditions. A reaction mechanism is proposed.

## Introduction

**Figure 1:**
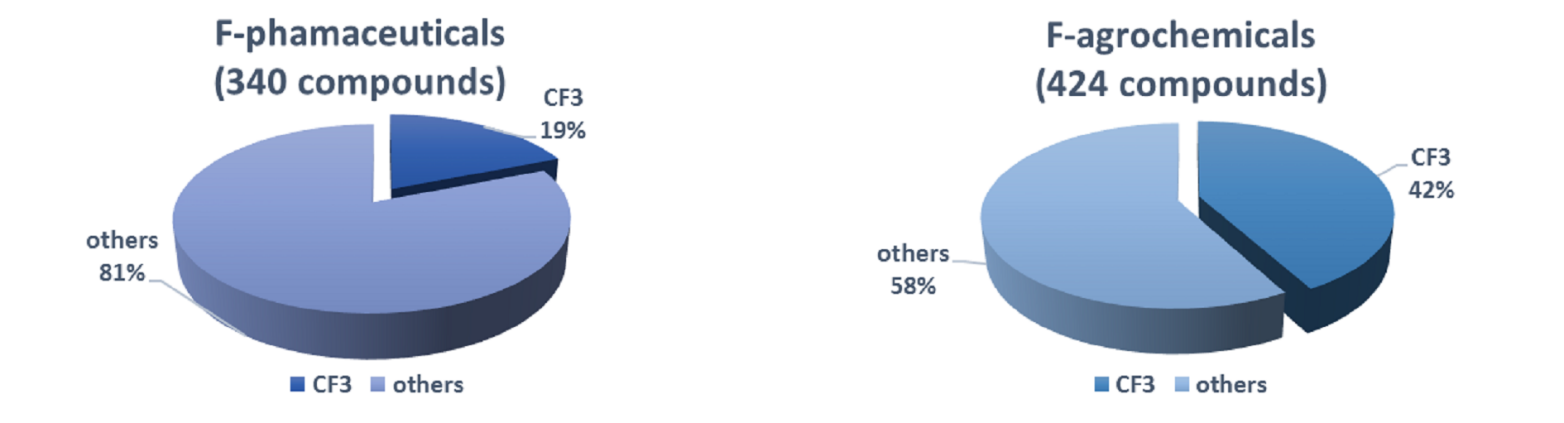
Ratios of CF_3_-containing drugs in marketed fluoro-pharmaceuticals and registered fluoro-agrochemicals in the world.

**Figure 2:**
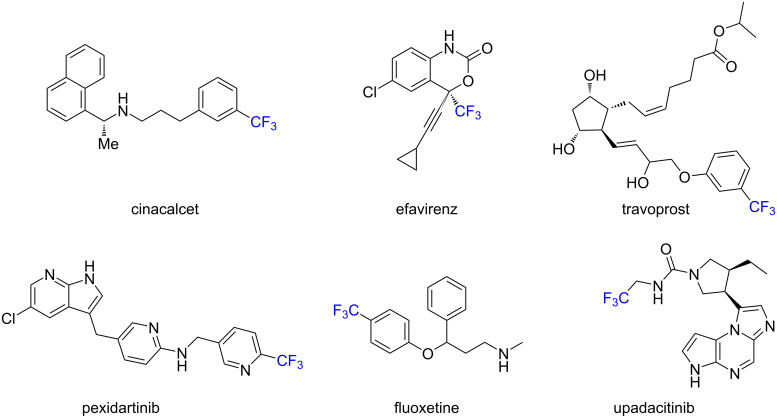
Selected examples of CF_3_-containing biologically active molecules.

Due to an impressively wide effect of fluorine on the biological activity, the insertion of fluorine atoms or fluorine-containing functional groups into organic molecules has become a common strategy in pharmaceutical and agrochemical industries [1–5]. Among various fluorine-containing functional groups, the trifluoromethyl (CF_3_) group has received countless attention in the design of novel drugs [6–7]. One utility of the CF_3_ group is the replacement of a methyl group in biologically active molecules to avoid the metabolic oxidation of a reactive methyl group in the parent molecules [8]. It should be noted that 19% out of 340 marketed fluoro-pharmaceuticals [4] and 42% out of 424 registered fluoro-agrochemicals [5] contain a CF_3_ moiety in their structures (Figure 1), but this motif has never been found in nature [9]. Several notable examples of CF_3_-containing pharmaceuticals and agrochemicals include cinacalcet, efavirenz, travoprost, pexidartinib, fluoxetine, and upadacitinib (Figure 2) [4–5].

Methodologies for the synthesis of trifluoromethyl compounds can be modestly divided into two general categories: the direct introduction of a CF_3_ group into the target position by trifluoromethylation [10–11], and the construction of a CF_3_ unit by transforming other functional groups [12–13]. Our group has developed various efficient methodologies for the electrophilic [14–15], nucleophilic [16], and radical [17] trifluoromethylation reactions for more than a decade. In recent years, we also reported the direct introduction of an acyl fluoride unit into aromatic compounds by the Pd-catalyzed cross-coupling reaction of aryl, vinyl, and heteroaryl iodides with 2-(difluoromethoxy)-5-nitropyridine [18]. A wide variety of acyl fluorides were efficiently obtained in high yields. We were fascinated by the synthetic versatility of acyl fluorides [19] to form other functional groups during our research on acyl fluorides. Acyl fluorides, which are one type of acyl halides, show distinct stability in the presence of moisture, indicating their suitable reactivity only in selected conditions [19].

**Scheme 1:**
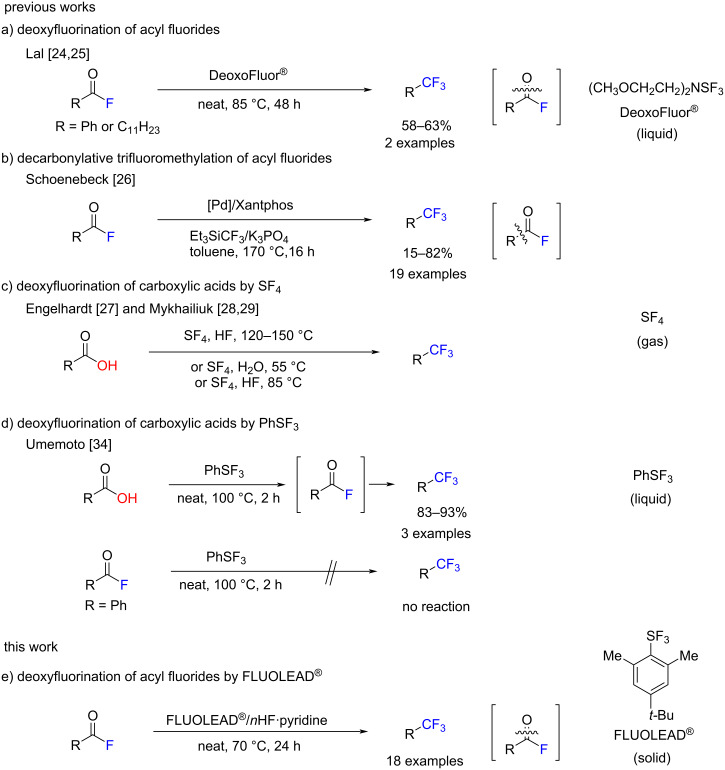
Transformation of acyl fluorides to trifluoromethyl compounds. a) Deoxyfluorination of acyl fluorides by DeoxoFluor^®^. b) Decarbonylative trifluoromethylation of acyl fluorides by Et_3_SiCF_3_. c) Deoxyfluorination of carboxylic acids by SF_4_. d) Deoxyfluorination of carboxylic acids by PhSF_3_ via acyl fluoride intermediates. e) Deoxyfluorination of acyl fluorides by FLUOLEAD^®^.

In addition to our direct cross-coupling reaction method, several useful synthetic methods for the formation of acyl fluorides have become available in recent years [19–23]. In this context, we were interested in the functional transformation of an acyl fluoride unit into a CF_3_ motif. Despite the current rich availability of acyl fluorides and a strong market demand for trifluoromethyl compounds, synthetic methods for the direct transformation of acyl fluorides to trifluoromethyl compounds are rare [24–26]. A seminal example is the work by Lal and co-workers reported in 1999 (Scheme 1a) [24–25]. The acyl fluorides were converted into the trifluoromethyl compounds in good yields using Deoxo-Fluor^®^, but they only provided two examples. In 2018, Schoenebeck and co-workers reported the decarbonylative trifluoromethylation of acyl fluorides by trifluoromethyl triethylsilane (Et_3_SiCF_3_) under Pd catalysis at high temperature (Scheme 1b) [26], although the reaction was categorized as trifluoromethylation and not as fluorination of acyl fluorides. Thus, the acyl fluoride moiety is commonly sacrificed as a “leaving group” in reactions. On the other hand, the transformation to CF_3_ derivatives from carboxylic acids are traditionally examined. In 1960, Engelhardt [27] performed the deoxyfluorination of carboxylic acids with sulfur tetrafluoride (SF_4_) at 120 to 150 °C in liquid hydrogen fluoride (HF) (Scheme 1c). Very recently, Mykhailiuk [28–29] improved the method to use SF_4_ for the deoxofluorination of carboxylic acids with or without HF in the gram scale (Scheme 1c). Although Mykhailiuk’s protocol is beneficial even for industrial applications, the use of toxic SF_4_ requires special apparatus with expert handling.

In 2010, Umemoto and co-workers developed a novel deoxyfluorinating agent, FLUOLEAD^®^ [30]. FLUOLEAD^®^ is a nucleophilic fluorinating agent that can be used as a broad and general substrate for the deoxyfluorination. FLUOLEAD^®^ is a stable crystalline solid and has sufficient stability against moisture to be handled open to air without the emission of fumes, and it is available in quantities that can exceed 100 kg from Ube Industries, Ltd [31]. FLUOLEAD^®^ is an attractive alternative to the toxic sulfur tetrafluoride [32] and the explosive (diethylamino)sulfur trifluoride (DAST) [33]. Although the 2010 report by Umemoto provided a method for the deoxyfluorination of a wide variety of substrates, including alcohols, ketones, and even carboxylic acids to the mono-, di-, and trifluoromethyl compounds using FLUOLEAD^®^ [30], they did not show the direct transformation of an acyl fluoride moiety to a CF_3_ unit. In 2012, Umemoto and Singh examined the deoxyfluorination of carboxylic acids to trifluoromethyl compounds by phenylsulfur trifluoride (PhSF_3_) [34]. They suggested that the transformation of carboxylic acids to the trifluoromethyl units consisted of two steps, including the generation of acyl fluoride intermediates (Scheme 1d). However, the direct transformation of acyl fluorides to trifluoromethyl compounds by PhSF_3_ did not occur. They explained the importance of the HF generation in the first step from acids to acyl fluorides and for the second step the transformation of the acyl fluorides to the trifluoromethyl compounds. Others have also suggested the critical role of HF for transforming the carboxylic acids to the trifluoromethyl compounds [27–29,35]. Based on these facts, we herein report the efficient deoxyfluorination protocol of acyl fluorides to the trifluoromethyl compounds using FLUOLEAD^®^ (Scheme 1e). The key to the successful transformation is the use of FLUOLEAD^®^ combined with Olah’s reagent [36] in solvent-free conditions. A wide variety of acyl fluorides involving aryloyl, (heteroaryl)oyl, and even aliphatic acyl, as well as some drug-like molecules, were smoothly transformed into the corresponding trifluoromethyl compounds in good to excellent yields by FLUOLEAD^®^/Olah’s reagent. Since both FLUOLEAD^®^ and Olah’s reagent are commercially available in industrial quantities, this would be an ideal method for the synthesis of trifluoromethyl compounds.

## Results and Discussion

First, [1,1'-biphenyl]-4-carbonyl fluoride (**1a**) was selected as the benchmark substrate to optimize the reaction conditions (see Table S1 in Supporting Information File 1 for an extensive list of reaction conditions). The investigation of a range of parameters showed that the best results were achieved by the combination of 3 equiv FLUOLEAD^®^ and 5 equiv *n*HF·pyridine in solvent-free conditions at 70 °C for 24 h, providing the product 4-(trifluoromethyl)-1,1'-biphenyl (**2a**) in 99% ^19^F NMR yield (Table 1, entry 1). Control experiments conducted in the absence of either FLUOLEAD^®^ or *n*HF·pyridine resulted in no desired product **2a** (Table 1, entries 2 and 3). Moreover, other fluorinating agents, such as DAST, DeoxoFluor, Xtalfluor-M^®^, and tetrabutylammonium difluorotriphenylsilicate (TBAT), were less effective (Table 1, entry 4). When NEt_3_·(HF)_3_ was used instead of *n*HF·pyridine, the yield decreased to 37% (Table 1, entry 5). Using a solvent to participate in the reaction reduced the reaction yield or even resulted in no reaction (Table 1, entry 6). Testing different reaction times indicated that 24 h were necessary (Table 1, entry 7). Lowering the temperature resulted in a decrease in the solubility of substrate **1a**, while raising the temperature to 100 °C allowed a similar yield to be obtained (Table 1, entry 8).

**Table 1:** Optimizing the reaction conditions for the conversion of **1a**^a^.

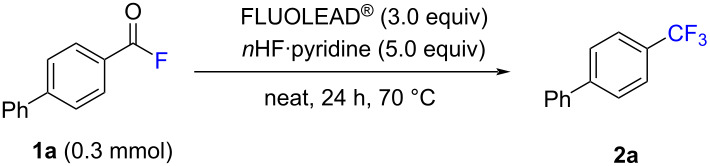		

entry	deviation from the standard conditions	yield (%)^b^

1	none	>99 (91)
2	no FLUOLEAD^®^	0
3	no *n*HF·pyridine	0
4	using DAST, DeoxoFluor, Xtalfluor-M, TBAT instead of FLUOLEAD^®^	25, 0, 0, 0
5	using NEt_3_·(HF) _3_ instead of *n*HF·pyridine	37
6	using MeCN, DMF, THF, toluene, or DCM, instead of neat	0, 0, 0, 65,26
7	using 17 h, 3 h or 1 h instead 24 h	55, 22, 9
8	using 40 °C, 50 °C, 100 °C instead of 70 °C	9, 68, 85

^a^Standard conditions: **1a** (0.3 mmol), FLUOLEAD^®^ (0.9 mmol) and *n*HF·pyridine (1.5 mmol) in neat conditions at 70 °C for 24 h. ^b^Determined by ^19^F NMR spectroscopy; the number in parentheses refers to the isolated yield.

**Scheme 2:**
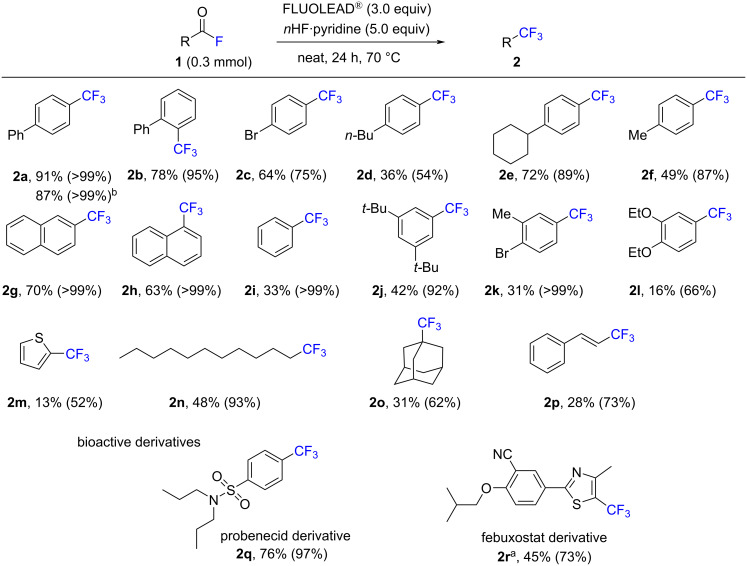
The substrate scope of acyl fluorides. Reaction conditions: **1** (0.3 mmol), FLUOLEAD^®^ (0.9 mmol, 3.0 equiv) and *n*HF·pyridine (1.5 mmol, 5.0 equiv) in neat conditions at 70 °C for 24 h. Yields in parentheses were determined by ^19^F NMR spectroscopy. ^a^At 100 °C. ^b^Using **1a** (1.0 g, 5.0 mmol).

With the optimized conditions in hand, the reaction scope was explored, and the results are summarized in Scheme 2. Various acyl fluorides were investigated in the presence of 3 equiv of FLUOLEAD^®^ and 5 equiv of nHF·pyridine. We first examined the deoxyfluorination of biarylacyl fluorides **1a** and **1b** with a *para*- or *ortho*-substitution. Both substrates gave excellent yields of the desired product **2** (95–99%). Phenyl substrates with a functional group, such as bromo, butyl, cyclohexyl, and methyl at the *para*-position afforded the desired products **2c**–**f** in good to high yields (54–89%). Naphthoyl fluorides **1g** and **1h** gave the desired products **2g** and **2h** in excellent yields (99%). Benzoyl fluoride (**1i**) also furnished trifluoromethylbenzene (**2i**) in an excellent yield (99%). Substrates with two substituents, such as 3,5-dibutyl, 1-bromo-2-methyl, and 1,2-diethoxy groups (**1j**–**l**), furnished the desired products **2j**–**l** in good to excellent yields (66–99%). The heteroaryl group of 2-thiophene (**1m**), the alkyl substrates (**1n** and **1o**) and vinylacyl fluoride (**1p**) were attempted and gave the desired products **2m**–**p** in good to excellent yields (52–93%). Moreover, drug-like derivatives were next investigated as substrates to assess further the utility of this method. The acyl fluoride of the probenecid derivative **1q** was successfully transformed into the corresponding trifluoromethyl compound in an excellent yield of 97% under the same conditions. The deoxyfluorination of the acyl fluoride in febuxostat (**1r**) required a higher reaction temperature (100 °C), and gave the corresponding CF_3_ product **2r** in a 73% yield. The gram-scale transformation of **1a** to **2a** was also performed under the same conditions providing a similar result (87% isolated yield). In all cases, the yields of the CF_3_ products **2** were high to excellent, as determined by NMR, while the isolated yields decreased due to the high volatility of the products **2**.

**Scheme 3:**
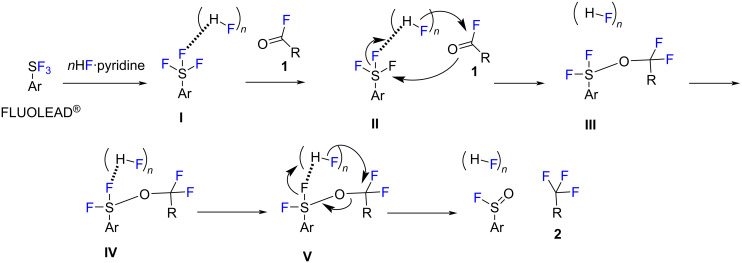
Mechanism of deoxyfluorination of acyl fluorides **1** with FLUOLEAD^®^/Olah’s reagent to trifluoromethyl compounds **2**.

A mechanism of the deoxyfluorination of the acyl fluorides with FLUOLEAD^®^/*n*HF·pyridine is proposed in Scheme 3. First, FLUOLEAD^®^ is activated with (HF)*n* via hydrogen bonding to provide an activated form **I**, which induces a nucleophilic attack from the carbonyl oxygen of **1** to **I** providing an intermediate **III** via transition state **II**. The intermediate **III** is further activated by (HF)*n* to form **IV**, which finally induces the deoxyfluorination via **V** furnishing the trifluoromethylated products **2** with the release of ArS(O)F and (HF)*n*.

## Conclusion

We have reported an efficient protocol for the deoxyfluorination of acyl fluoride substrates, using FLUOLEAD^®^ as the deoxofluorinating agent in the presence of Olah’s reagent, to generate trifluoromethyl compounds. FLUOLEAD^®^ combined with Olah’s reagent, *n*HF·pyridine, was most effective for this functional transformation reaction. The reaction of acyl fluorides afforded the desired products smoothly in good to high yield in solvent-free conditions. An extension of this deoxyfluorination strategy to drug-like molecules was demonstrated to show the usefulness of this transformation. The present protocol also expands the utility of FLUOLEAD^®^ in organic synthesis.

## Experimental

General procedure: An oven-dried narrow-mouth FEP tube (Nalgene^®^, 10.0 mL) containing a magnetic stirring bar was charged with substrate **1** (0.3 mmol), FLUOLEAD^®^ (225.3 mg, 0.9 mmol, 3.0 equiv) and the *n*HF·pyridine complex (HF 70%, pyridine 30%, 1.5 mmol, 5.0 equiv, neat). The tube was tightly sealed and the reaction mixture stirred at 70 °C for 24 h. Then the mixture was cooled to room temperature and ethanol (3.0 mL) was added to the mixture while stirring for an additional 30 min at room temperature. The mixture was then added to 1 M aqueous NaHCO_3_ (3 mL) at 0 °C, extracted with Et_2_O (3 × 5 mL), and the combined organic layer was dried over anhydrous Na_2_SO_4_, filtered, and concentrated under reduced pressure. The yield was determined by ^19^F NMR analysis of the crude mixture by using C_6_H_5_OCF_3_ (40.0 μL, 0.3 mmol, 1.0 equiv) as an internal standard. The residue was purified by silica gel flash chromatography (*n*-hexane) to afford the title compounds.

## Supporting Information

File 1 Optimization of the reaction conditions, general procedure and product characterization data.
