# Supplementary material for: Deoxyfluorination of acyl fluorides to trifluoromethyl compounds by FLUOLEAD®/Olah’s reagent under solvent-free conditions
Source: Beilstein J Org Chem. 2020 Dec 14;16:3052–8. doi: 10.3762/bjoc.16.254 (PMC7753111; doi:10.3762/bjoc.16.254)
Supplement: File 1 — Optimization of the reaction conditions, general procedure and product characterization data. [file Beilstein_J_Org_Chem-16-3052-s001.pdf]

BEILSTEIN JOURNAL OF ORGANIC CHEMISTRY

Supporting Information

for

Deoxyfluorination of acyl fluorides to trifluoromethyl compounds by FLUOLEAD®/Olah's reagent under solvent-free conditions

Yumeng Liang, Akihito Taya, Zhengyu Zhao, Norimichi Saito and Norio Shibata

Beilstein J. Org. Chem. **2020,** *16,* 3052–3058. doi:10.3762/bjoc.16.254

Optimization of the reaction conditions, general procedure and product characterization data

License and Terms: This is a supporting information file under the terms of the Creative Commons Attribution License (https://creativecommons.org/licenses/by/4.0). Please note that the reuse, redistribution and reproduction in particular requires that the author(s) and source are credited and that individual graphics may be subject to special legal provisions.
The license is subject to the *Beilstein Journal of Organic Chemistry* terms and conditions: (https://www.beilstein-journals.org/bjoc/terms)

1. General Information.

All reagents were used as received from commercial sources, unless specified otherwise. All reactions were performed in narrow-mouth FEP tube (Nalgene®). All reactions were monitored by thin-layer chromatography (TLC) carried out on 0.25 mm Merck silica-gel (60-F254). The TLC plates were visualized with UV light (254 nm) and potassium permanganate or *p*-anisaldehyde in ethanol/heat. Purification of reaction products was carried out by column chromatography with silica-gel 60N spherical neutral size 63-210 μm or 40-63 μm. Unless otherwise specified, the ^{1}H-NMR (300 MHz and 700 MHz), ^{19}F-NMR (282 MHz), ^{13}C-NMR (90 MHz, 126 MHz and 176 MHz) spectra for solution in $CDCl_3$ were recorded on a Buruker Avance 500, Varian Mercury 300, JEOL ECZ700R. Chemical shifts (δ) are expressed in ppm downfield from tetramethylsilane (δH = 0.00 ppm) or $CDCl_3$ (δC = 77.0 ppm) or hexafluorobenzene (δF = −162.20 ppm). Mass spectra was recorded on a JEOL JMS-Q1050GC Master-Quad GC/MS (EI-MS) and SHIMADZU LCMS-2020 (ESI-MS). High resolution mass spectrometry (HRMS) was recorded on a Waters, GCT Premier (EI-MS) with a TOF analyzer and Waters Synapt G2 HDMS (ESI-MS). The wave numbers (ν) of recorded IR-signals are quoted in cm^{-1} on a JASCO FT/IR-4100 spectrometer. Melting point was recorded on a BUCHI M-565. All solvents were dried and distilled before use.

2. Optimization of Reaction Conditions.

Table S1. Optimization of reaction condition with **2a**.

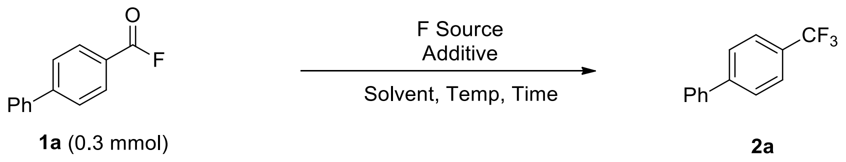

Entry	[F] Source (x equiv)	Additive (y equiv)	Solvent (z mL)	Temp. (°C)	Time (h)	Yield (%)[a]
1	DAST (3)	-	MeCN (1)	RT	24	ND
2	Fluolead (3)	-	MeCN (1)	RT	24	ND
3	nHF·pyridine (2)	-	MeCN (1)	RT	24	ND
4	DAST (3)	-	MeCN (1)	80	24	3
5	Fluolead (3)	-	MeCN (1)	80	24	ND
6	nHF·pyridine (2)	-	MeCN (1)	80	24	ND
7	DAST (3)	-	MeCN (1)	80	48	25
8	Fluolead (3)	nHF·pyridine (3)	neat	70	24	75
9	Fluolead (3)	nHF·pyridine (1)	neat	70	24	26
10	Fluolead (3)	nHF·pyridine (2)	neat	70	24	20
11	Fluolead (3)	nHF·pyridine (4)	neat	70	24	90
12	Fluolead (3)	nHF·pyridine (5)	neat	70	24	>99(91)
13	Fluolead (3)	-	neat	70	24	ND
14	Fluolead (2)	nHF·pyridine (5)	neat	70	24	65
15[b]	Fluolead (1.5)	nHF·pyridine (5)	neat	70	24	63
16	Fluolead (1)	nHF·pyridine (5)	neat	70	24	53
17	Fluolead (0.7)	nHF·pyridine (5)	neat	70	24	36
18	-	nHF·pyridine (5)	neat	70	24	ND
19	Fluolead (3)	$Et_3N(HF)_3$ (5)	neat	70	24	37
20	DAST (3)	nHF·pyridine (5)	neat	70	24	25
21	DeoxoFluor (3)	nHF·pyridine (5)	neat	70	24	ND
22	Xtalfluor-M (3)	nHF·pyridine (5)	neat	70	24	ND
23	TBAT (3)	nHF·pyridine (5)	neat	70	24	ND
24	Fluolead (3)	nHF·pyridine (5)	MeCN (1)	70	24	ND

25	Fluolead (3)	nHF·pyridine (5)	DMF (1)	70	24	ND
26	Fluolead (3)	nHF·pyridine (5)	THF (1)	70	24	ND
27	Fluolead (3)	nHF·pyridine (5)	toluene (1)	70	24	65
28	Fluolead (3)	nHF·pyridine (5)	DCM (1)	70	24	26
29	Fluolead (3)	nHF·pyridine (5)	neat	70	17	55
30	Fluolead (3)	nHF·pyridine (5)	neat	70	3	22
31	Fluolead (3)	nHF·pyridine (5)	neat	70	1	9
32	Fluolead (3)	nHF·pyridine (5)	neat	40	24	9
33	Fluolead (3)	nHF·pyridine (5)	neat	50	24	68
34	Fluolead (3)	nHF·pyridine (5)	neat	100	24	85

Standard conditons:**1a** (0.3 mmol), [F] source and Additive in solvent or neat. [a] Determined by ^{19}F NMR spectroscopy. The numbers in parentheses refer to the isolated yield.

3. General Procedure and Product Characterization Data for Trifluoromethyl Compounds 2.

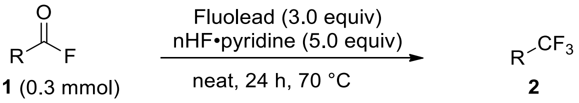

Scheme S1. General procedure for trifluoromethyl compounds **2** from acyl fluoride **1.**

General procedure:

An oven-dried narrow-mouth FEP tube (Nalgene®) (10.0 mL) containing a magnetic stir bar was charged with **1** (0.3 mmol), Fluolead (225.3 mg, 0.9 mmol, 3.0 equiv.) and nHF·Pyridine complex (HF 70%, pyridine 30%, 1.5 mmol, 5.0 equiv., neat). The tube was tightly sealed then stirred at 70 °C for 24 hours. Then, the mixtures were cooled down to room temperature and ethanol (3.0 mL) was added to the mixtures stirring for additional 30 min at room temperature. Then, the mixtures were added to 1 M aqueous $NaHCO_3$ (3 mL) at 0°C, extracted with Et_2O (3 x 5mL) and the combined organic layer was dried over anhydrous Na_2SO_4, filtered and concentrated under reduced pressure. The yield was determined by ^{19}F NMR analysis of the crude mixture by using $C_6H_5OCF_3$ (40.0 μL, 0.3 mmol, 1.0 equiv.) as an internal standard. Unless specifically mentioned, the residue was purified by silica gel flash chromatography (*n*-Hexane) to afford the title compound.

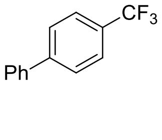

4-(Trifluoromethyl)-1,1'-biphenyl (2a).

Following the general procedure, using substrate **1a** (0.3 mmol, 1.0 equiv), Fluolead (225.3 mg, 0.9 mmol, 3.0 equiv.) and nHF·Pyridine complex (HF 70%, pyridine 30%, 1.5 mmol, 5.0 equiv., neat), the reaction mixture was stirred at 70 °C for 24 hours. The yield (>99%) was determined by ^{19}F NMR of the crude reaction mixture.

The crude mixture was purified by column chromatography (*n*-Hexane) to afford the title compound **2a** (60.7 mg, 91% yield) as a white solid.

^{1}H NMR (300 MHz, $CDCl_3$) δ: 7.70 – 7.69 (m, 4H), 7.62 – 7.58 (m, 2H), 7.50 – 7.38 (m, 3H) ppm. **^{19}F NMR** (282 MHz, $CDCl_3$) δ: -62.88 (s, 3F) ppm. **MS (EI)**: *m/z* 222 $[M]^+$. Spectroscopic data was agreement with the literature.[1]

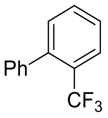

2-(Trifluoromethyl)-1,1'-biphenyl (2b).

Following the general procedure, using substrate **1b** (0.3 mmol, 1.0 equiv), Fluolead (225.3 mg, 0.9 mmol, 3.0 equiv.) and nHF·Pyridine complex (HF 70%, pyridine 30%, 1.5 mmol, 5.0 equiv., neat), the reaction mixture was stirred at 70 °C for 24 hours. The yield (95%) was determined by ^{19}F NMR of the crude reaction mixture. The crude mixture was purified by column chromatography (*n*-Hexane) to afford the title compound **2b** (52.0 mg, 78% yield) as a white solid.

^{1}H NMR (300 MHz, $CDCl_3$) δ: 7.75 (d, J = 7.6 Hz, 1H), 7.58 – 7.53 (m, 1H), 7.49 – 7.44 (m, 1H), 7.41–7.39 (m, 3H), 7.34 – 7.31 (m, 3H) ppm. **^{19}F NMR** (282 MHz, $CDCl_3$) δ: -57.3 (s, 3F) ppm. **MS (EI)**: *m/z* 222 $[M]^+$. Spectroscopic data was agreement with the literature.[1]

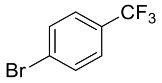

1-Bromo-4-(trifluoromethyl)benzene (2c).

Following the general procedure, using substrate **1c** (0.3 mmol, 1.0 equiv), Fluolead (225.3 mg, 0.9 mmol, 3.0 equiv.) and nHF·Pyridine complex (HF 70%, pyridine 30%, 1.5 mmol, 5.0 equiv., neat), the reaction mixture was stirred at 70 °C for 24 hours. The yield (75%) was determined by ^{19}F NMR of the crude reaction mixture. The crude mixture was purified by column chromatography (*n*-Hexane) to afford the title compound **2c** (43.1 mg, 64% yield) as a colorless oil.

^{1}H NMR (300 MHz, $CDCl_3$) δ: 7.64 (d, J = 7.3 Hz, 2H), 7.50 (d, J = 6.8 Hz, 2H) ppm. **^{19}F NMR** (282 MHz, $CDCl_3$) δ: -63.04 (s, 3F) ppm. **MS** (EI): m/z 224 $[M]^+$. Spectroscopic data was agreement with the literature.[2]

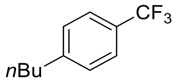

1-Butyl-4-(trifluoromethyl)benzene (2d).

Following the general procedure, using substrate **1d** (0.3 mmol, 1.0 equiv), Fluolead (225.3 mg, 0.9 mmol, 3.0 equiv.) and nHF·Pyridine complex (HF 70%, pyridine 30%, 1.5 mmol, 5.0 equiv., neat), the reaction mixture was stirred at 70 °C for 24 hours. The yield (54%) was determined by ^{19}F NMR of the crude reaction mixture. The crude mixture was purified by column chromatography (*n*-Hexane) to afford the title compound **2d** (21.8 mg, 36% yield) as a colorless oil.

^{1}H NMR (300 MHz, $CDCl_3$) δ: 7.52 (d, *J* = 8.0 Hz, 2H), 7.28 (d, *J* = 7.8 Hz, 2H), 2.66 (t, *J* = 7.7 Hz, 2H), 1.66 – 1.54 (m, 2H), 1.39 – 1.32 (m, 2H), 0.93 (t, *J* = 7.3 Hz, 3H) ppm. **^{19}F NMR** (282 MHz, $CDCl_3$) δ: -62.72 (s, 3F) ppm. **MS** (EI): m/z 202 $[M]^+$. Spectroscopic data was agreement with the literature. [3]

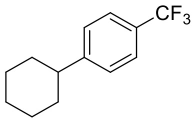

1-Cyclohexyl-4-(trifluoromethyl)benzene (2e).

Following the general procedure, using substrate **1e** (0.3 mmol, 1.0 equiv), Fluolead (225.3 mg, 0.9 mmol, 3.0 equiv.) and nHF·Pyridine complex (HF 70%, pyridine 30%, 1.5 mmol, 5.0 equiv., neat), the reaction mixture was stirred at 70 °C for 24 hours. The yield (89%) was determined by ^{19}F NMR of the crude reaction mixture. The crude mixture was purified by column chromatography (*n*-Hexane) to afford the title compound **2e** (49.1 mg, 72% yield) as a colorless oil.

^{1}H NMR (300 MHz, $CDCl_3$) δ: 7.54 (d, *J* = 8.0 Hz, 2H), 7.31 (d, *J* = 8.0 Hz, 2H), 2.60 – 2.52 (m, 1H), 1.89 – 1.84 (m, 4H), 1.79 – 1.74 (m, 1H), 1.49 – 1.38(m, 4H), 1.33 – 1.22(m, 1H) ppm. **^{19}F NMR** (282 MHz, $CDCl_3$) δ: -62.73 (s, 3F) ppm. **MS** (EI): m/z 228 $[M]^+$. Spectroscopic data was agreement with the literature. [4]

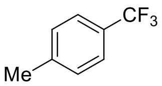

1-Methyl-4-(trifluoromethyl)benzene (2f).

Following the general procedure, using substrate **1f** (0.3 mmol, 1.0 equiv), Fluolead (225.3 mg, 0.9 mmol, 3.0 equiv.) and nHF·Pyridine complex (HF 70%, pyridine 30%, 1.5 mmol, 5.0 equiv., neat), the reaction mixture was stirred at 70 °C for 24 hours. The yield (87%) was determined by ^{19}F NMR of the crude reaction mixture. The crude mixture was purified by column chromatography (*n*-Hexane) to afford the title compound **2f** (23.6 mg, 49% yield) as a colorless oil.

^{1}H NMR (300 MHz, $CDCl_3$) δ: 8.19 (d, *J* = 8.2 Hz, 2H), 7.82 (d, *J* = 8.1 Hz, 2H), 2.41 (s, 3H) ppm. **^{19}F NMR** (282 MHz, $CDCl_3$) δ: -63.09 (s, 3F) ppm. **MS** (EI): m/z 192 $[M]^+$. Spectroscopic data was agreement with the literature. [1]

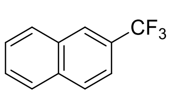

2-(Trifluoromethyl)naphthalene (2g).

Following the general procedure, using substrate **1g** (0.3 mmol, 1.0 equiv), Fluolead (225.3 mg, 0.9 mmol, 3.0 equiv.) and nHF·Pyridine complex (HF 70%, pyridine 30%, 1.5 mmol, 5.0 equiv., neat), the reaction mixture was stirred at 70 °C for 24 hours. The yield (>99%) was determined by ^{19}F NMR of the crude reaction mixture. The crude mixture was purified by column chromatography (*n*-Hexane) to afford the title compound **2g** (41.2 mg, 70% yield) as a white solid.

^{1}H NMR (300 MHz, $CDCl_3$) δ: 8.16 (s, 1H), 7.97 – 7.90 (m, 3H), 7.66 – 7.56 (m, 3H) ppm. **^{19}F NMR** (282

MHz, $CDCl_3$) δ: -62.76 (s, 3F) ppm. **MS** (EI): m/z 196 $[M]^+$. Spectroscopic data was agreement with the literature. [2]

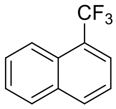

1-(Trifluoromethyl)naphthalene (2h).

Following the general procedure, using substrate **1h** (0.3 mmol, 1.0 equiv), Fluolead (225.3 mg, 0.9 mmol, 3.0 equiv.) and nHF·Pyridine complex (HF 70%, pyridine 30%, 1.5 mmol, 5.0 equiv., neat), the reaction mixture was stirred at 70 °C for 24 hours. The yield (>99%) was determined by ^{19}F NMR of the crude reaction mixture. The crude mixture was purified by column chromatography (*n*-Hexane) to afford the title compound **2h** (36.8 mg, 63% yield) as a colorless oil.

^{1}H NMR (300 MHz, $CDCl_3$) δ: 8.20 (d, J = 8.5 Hz, 1H), 8.03 (d, J = 8.4 Hz, 1H), 7.90 (q, J = 15.9, 7.5 Hz, 2H), 7.66 – 7.48 (m, 3H) ppm. **^{19}F NMR** (282 MHz, $CDCl_3$) δ: -60.25 (s, 3F) ppm. **MS** (EI): m/z 196 $[M]^+$. Spectroscopic data was agreement with the literature. [1]

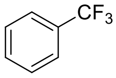

(Trifluoromethyl)benzene (2i).

Following the general procedure, using substrate **1i** (0.3 mmol, 1.0 equiv), Fluolead (225.3 mg, 0.9 mmol, 3.0 equiv.) and nHF·Pyridine complex (HF 70%, pyridine 30%, 1.5 mmol, 5.0 equiv., neat), the reaction mixture was stirred at 70 °C for 24 hours. The yield (>99%) was determined by ^{19}F NMR of the crude reaction mixture. The crude mixture was purified by kugelrohr distillation to afford the title compound **2i** (14.5 mg, 33% yield) as a colorless oil.

^{1}H NMR (300 MHz, $CDCl_3$) δ: 7.64 – 7.61 (m, 2H), 7.58 – 7.45 (m, 3H). **^{19}F NMR** (282 MHz, $CDCl_3$) δ: -63.20 (s, 3F) ppm. **MS** (EI): m/z 146 $[M]^+$. Spectroscopic data was agreement with the literature. [5]

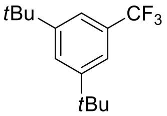

1,3-Di-tert-butyl-5-(trifluoromethyl)benzene (2j).

Following the general procedure, using substrate **1j** (0.3 mmol, 1.0 equiv), Fluolead (225.3 mg, 0.9 mmol, 3.0 equiv.) and nHF·Pyridine complex (HF 70%, pyridine 30%, 1.5 mmol, 5.0 equiv., neat), the reaction mixture was stirred at 70 °C for 24 hours. The yield (92%) was determined by ^{19}F NMR of the crude reaction mixture. The crude mixture was purified by column chromatography (*n*-Hexane) to afford the title compound **2j** (32.8 mg, 42% yield) as a white solid.

^{1}H NMR (300 MHz, $CDCl_3$) δ: 7.59 (s, 1H), 7.44 (s, 2H), 1.34 (s, 18H) ppm. **^{19}F NMR** (282 MHz, $CDCl_3$) δ: -62.60 (s, 3F) ppm. **^{13}C NMR** (176 MHz, $CDCl_3$) δ: 151.60, 129.91 (q, J = 31.1 Hz), 124.71 (q, J = 272.2 Hz) 125.79, 119.27 (q, J = 3.3 Hz), 35.02, 31.30 ppm. **^{19}F NMR** (282 MHz, $CDCl_3$) δ: -62.60 (s, 3F) ppm. **HRMS**

(EI) calculated for $C_{15}H_{21}F_3$ $[M]^+$: 258.1595 found 258.1601. **IR (KBr)**: 2967, 2869, 1477, 1342, 1261, 1126, 884 cm^{-1}. **m.p.**: 36.2 – 36.9 °C.

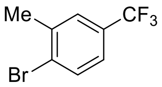

1-Bromo-2-methyl-4-(trifluoromethyl)benzene (2k).

Following the general procedure, using substrate **1k** (0.3 mmol, 1.0 equiv), Fluolead (225.3 mg, 0.9 mmol, 3.0 equiv.) and nHF·Pyridine complex (HF 70%, pyridine 30%, 1.5 mmol, 5.0 equiv., neat), the reaction mixture was stirred at 70 °C for 24 hours. The yield (>99%) was determined by ^{19}F NMR of the crude reaction mixture. The crude mixture was purified by column chromatography (*n*-Hexane) to afford the title compound **2k** (22.0 mg, 31% yield) as a colorless oil.

^{1}H NMR (300 MHz, $CDCl_3$) δ: 7.65 (d, *J* = 8.4 Hz, 1H), 7.49 (s, 1H), 7.31 (d, *J* = 8.5 Hz, 1H), 2.46 (s, 3H) ppm. **^{19}F NMR** (282 MHz, $CDCl_3$) δ: -63.17 (s, 3F) ppm. **MS** (EI): m/z 238 $[M]^+$. Spectroscopic data was agreement with the literature. [6]

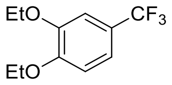

1,2-Diethoxy-4-(trifluoromethyl)benzene (2l).

Following the general procedure, using substrate **1l** (0.3 mmol, 1.0 equiv), Fluolead (225.3 mg, 0.9 mmol, 3.0 equiv.) and nHF·Pyridine complex (HF 70%, pyridine 30%, 1.5 mmol, 5.0 equiv., neat), the reaction mixture was stirred at 70 °C for 24 hours. The yield (66%) was determined by ^{19}F NMR of the crude reaction mixture. The crude mixture was purified by column chromatography (*n*-Hexane/ Et_2O = 5/1) to afford the title compound **2l** (10.9 mg, 16% yield) as a colorless oil.

^{1}H NMR (300 MHz, $CDCl_3$) δ: 7.17 (d, *J* = 8.8 Hz, 1H), 7.08 (s, 1H), 6.91 (d, *J* = 8.4 Hz, 1H), 4.13 (qd, *J* = 7.0, 3.7 Hz, 4H), 1.48 (t, *J* = 7.0 Hz, 6H) ppm. **^{19}F NMR** (282 MHz, $CDCl_3$) δ: -62.00 (s, 3F) ppm. **MS** (EI): m/z 234 $[M]^+$. Spectroscopic data was agreement with the literature. [7]

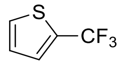

2-(Trifluoromethyl)thiophene (2m).

Following the general procedure, using substrate **1m** (0.3 mmol, 1.0 equiv), Fluolead (225.3 mg, 0.9 mmol, 3.0 equiv.) and nHF·Pyridine complex (HF 70%, pyridine 30%, 1.5 mmol, 5.0 equiv., neat), the reaction mixture was stirred at 70 °C for 24 hours. The yield (52%) was determined by ^{19}F NMR of the crude reaction mixture. The crude mixture was purified by kugelrohr distillation to afford the title compound **2m** (6.0 mg, 13% yield) as a colorless oil.

^{1}H NMR (300 MHz, $CDCl_3$) δ: 7.51 (d, *J* = 5.1 Hz, 1H), 7.46 (d, *J* = 3.4 Hz, 1H), 7.09 (s, 1H) ppm. **^{19}F NMR** (282 MHz, $CDCl_3$) δ: -55.44 (s, 3F) ppm. **MS** (EI): m/z 152 $[M]^+$. Spectroscopic data was agreement with the literature. [1]

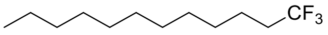

1,1,1-Trifluorododecane (2n).

Following the general procedure, using substrate **1n** (0.3 mmol, 1.0 equiv), Fluolead (225.3 mg, 0.9 mmol, 3.0 equiv.) and nHF·Pyridine complex (HF 70%, pyridine 30%, 1.5 mmol, 5.0 equiv., neat), the reaction mixture was stirred at 70 °C for 24 hours. The yield (93%) was determined by ^{19}F NMR of the crude reaction mixture. The crude mixture was purified by column chromatography (*n*-Hexane) to afford the title compound **2n** (32.3 mg, 48% yield) as a colorless oil.

^{1}H NMR (300 MHz, $CDCl_3$) δ: 2.12 – 2.01 (m, 2H), 1.60 – 1.50 (m, 2H), 1.30 – 1.27 (m, 16H), 0.92 – 0.86 (m, 3H) ppm. **^{19}F NMR** (282 MHz, $CDCl_3$) δ: -66.91 (s, 3F) ppm. **MS** (EI): m/z 224 $[M]^+$. Spectroscopic data was agreement with the literature. [8]

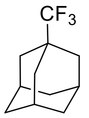

1-Trifluoromethyladamantane (2o).

Following the general procedure, using substrate **1o** (0.3 mmol, 1.0 equiv), Fluolead (225.3 mg, 0.9 mmol, 3.0 equiv.) and nHF·Pyridine complex (HF 70%, pyridine 30%, 1.5 mmol, 5.0 equiv., neat), the reaction mixture was stirred at 70 °C for 24 hours. The yield (62%) was determined by ^{19}F NMR of the crude reaction mixture. The crude mixture was purified by column chromatography (*n*-Hexane) to afford the title compound **2o** (19.0 mg, 31% yield) as a colorless oil.

^{1}H NMR (300 MHz, $CDCl_3$) δ: 2.06 – 2.04 (m, 3H), 1.78 – 1.65 (m, 12H) ppm. **^{19}F NMR** (282 MHz, $CDCl_3$) δ: -83.49 (s, 3F) ppm. **MS** (EI): m/z 204 $[M]^+$. Spectroscopic data was agreement with the literature. [9]

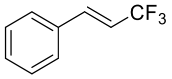

(*E*)-1-Phenyl-3,3,3-trifluoroprop-1-ene (2p).

Following the general procedure, using substrate **1p** (0.3 mmol, 1.0 equiv), Fluolead (225.3 mg, 0.9 mmol, 3.0 equiv.) and nHF·Pyridine complex (HF 70%, pyridine 30%, 1.5 mmol, 5.0 equiv., neat), the reaction mixture was stirred at 70 °C for 24 hours. The yield (73%) was determined by ^{19}F NMR of the crude reaction mixture. The crude mixture was purified by column chromatography (*n*-Hexane) to afford the title compound **2p** (14.5 mg, 28% yield) as a colorless oil.

^{1}H NMR (300 MHz, $CDCl_3$) δ: 7.48 – 7.43 (m, 2H), 7.40 – 7.38 (m, 3H), 7.15 (dq, J = 16.2, 2.0 Hz, 1H), 6.21 (dq, J = 16.1, 6.5 Hz, 1H) ppm. **^{19}F NMR** (282 MHz, $CDCl_3$) δ: -63.81 (d, J = 6.4 Hz, 3F) ppm. **MS** (EI): m/z 172 $[M]^+$. Spectroscopic data was agreement with the literature. [10]

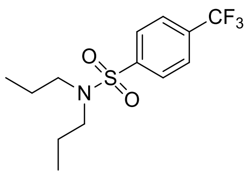

***N,N*-Dipropyl-4-(trifluoromethyl)benzenesulfonamide (2q).**

Following the general procedure, using substrate **1q** (0.3 mmol, 1.0 equiv), Fluolead (225.3 mg, 0.9 mmol, 3.0 equiv.) and nHF·Pyridine complex (HF 70%, pyridine 30%, 1.5 mmol, 5.0 equiv., neat), the reaction mixture was stirred at 70 °C for 24 hours. The yield (97%) was determined by ^{19}F NMR of the crude reaction mixture. The crude mixture was purified by column chromatography (*n*-Hexane/EtOAc = 10/1) to afford the title compound **2q** (70.5 mg, 76% yield) as an orange solid.

^{1}H NMR (300 MHz, $CDCl_3$) δ: 7.94 (d, *J* = 8.3 Hz, 2H), 7.77 (d, *J* = 8.2 Hz, 2H), 3.11 (t, *J* = 7.8 Hz, 4H), 1.63 – 1.50 (m, 4H), 0.88 (t, *J* = 7.4 Hz, 6H) ppm. **^{19}F NMR** (282 MHz, $CDCl_3$) δ: -63.51 (s, 3F) ppm. **MS** (ESI): m/z 310 $[M + H]^+$. Spectroscopic data was agreement with the literature. [8]

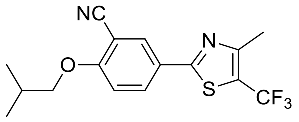

2-Isobutoxy-5-(4-methyl-5-(trifluoromethyl)thiazol-2-yl)benzonitrile (2r).

Following the general procedure, using substrate **1r** (0.3 mmol, 1.0 equiv), Fluolead (225.3 mg, 0.9 mmol, 3.0 equiv.) and nHF·Pyridine complex (HF 70%, pyridine 30%, 1.5 mmol, 5.0 equiv., neat), the reaction mixture was stirred at 100 °C for 24 hours. The yield (73%) was determined by ^{19}F NMR of the crude reaction mixture. The crude mixture was purified by column chromatography (*n*-Hexane/EtOAc = 10/1) to afford the title compound **2r** (45.9 mg, 45% yield) as a yellow solid.

^{1}H NMR (300 MHz, $CDCl_3$) δ: 8.14 (d, *J* = 1.7 Hz, 1H), 8.04 (dd, *J* = 8.8, 1.8 Hz, 1H), 7.02 (d, *J* = 8.9 Hz, 1H), 3.90 (d, *J* = 6.4 Hz, 2H), 2.59 (s, 3H), 2.25 – 2.17 (m, 1H), 1.11 (s, 3H), 1.08 (s, 3H) ppm. **^{13}C NMR** (126 MHz, $CDCl_3$) δ: 165.7, 162.5, 155.4 (q, *J* = 2.5 Hz), 132.4, 131.9, 125.7, 122.4 (q, *J* = 269.4 Hz), 119.7 (q, *J* = 37.3 Hz), 115.3, 112.6, 103.0, 75.7, 28.1, 19.0, 16.1 ppm. **^{19}F NMR** (282 MHz, $CDCl_3$) δ: -53.38 (s, 3F) ppm. **HRMS** (ESI) calculated for $C_{16}H_{16}N_2F_3OS$ $[M + H]^+$: 341.0935 found 341.0922. **IR** (KBr): 2960, 2881, 2224, 1612, 1514, 1459, 1382, 1349, 1279, 1169, 1121, 1020, 823, 444, 404 cm^{-1}. **m.p.**: 107.0-108.0 °C.

4. 1 Gram scale of 1a

An oven-dried narrow-mouth FEP tube (Nalgene®) (30.0 mL) containing a magnetic stir bar was charged with **1a** (1.0 g, 5.0 mmol), Fluolead (3.77 g, 15.0 mmol, 3.0 equiv.) and nHF·Pyridine complex (HF 70%, pyridine 30%, 25.0 mmol, 5.0 equiv., neat). The tube was tightly sealed then stirred at 70 °C for 24 hours. Then, the mixtures were cooled down to room temperature and menthol (10.0 mL) was added to the mixtures stirring for additional 30 min at room temperature. Then, the mixtures were added to 1 M aqueous $NaHCO_3$ (10.0 mL) at 0°C, extracted with Et_2O (3 x 20 mL) and the combined organic layer was dried over anhydrous Na_2SO_4, filtered and concentrated under reduced pressure. The yield (>99%) was determined by ^{19}F NMR analysis of

the crude mixture by using $C_6H_5OCF_3$ (515.0 µL, 5.0 mmol, 1.0 equiv.) as an internal standard. The crude mixture was purified by column chromatography (*n*-Hexane) to afford the title compound **2a** (963.1 mg, 87% yield) as a white solid.

5. Preparation of Starting Materials

Acyl fluoride **1i** is known compounds and obtained from commercial sources. Acyl fluorides **1a** – **1h, 1m** – **1r** were known compounds and synthesized according to the reported procedures.[11-14]

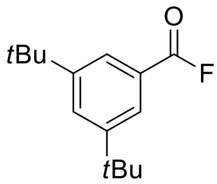

3,5-Di-*tert*-butylbenzoyl fluoride (1j).

To a solution of the corresponding carboxylic acid (3.0 mmol, 1.0 equiv) in Acetonitrile (10 mL) was added Fluolead (3.0 mmol, 1.0 equiv) at rt. The reaction mixture was stirred at rt for 2 hours, and then quenched with water. The resulting mixture was extracted with DCM (20 mL × 2). The combined organic layers were washed with brine, dried over anhydrous Na_2SO_4, filtered and concentrated under reduced pressure. The crude mixture was purified by column chromatography (*n*-Hexane) to afford the title compound **1j** (582.1 mg, 82% yield) as a white solid.

^{1}H NMR (300 MHz, $CDCl_3$) δ: 7.88 (s, 2H), 7.76 (s, 1H), 1.35 (s, 18H) ppm. **^{13}C NMR** (176 MHz, $CDCl_3$) δ: 156.7 (d, *J* = 342.2 Hz), 150.5, 128.2, 124.2, 122.9 (d, *J* = 62.6 Hz), 33.5, 29.8 ppm. **^{19}F NMR** (282 MHz, $CDCl_3$) δ: 17.44 (s, 1F) ppm. **HRMS** (EI) calculated for $C_{15}H_{21}FO$ $[M]^+$: 236.1576 found 236.1565. **IR** (KBr): 2960, 1808, 1475, 1217, 1062 cm^{-1}. **m.p.**: 81.8-82.3°C.

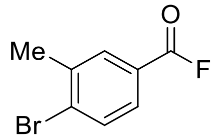

4-bromo-3-methylbenzoyl fluoride (1k).

To a solution of the corresponding carboxylic acid (3.0 mmol, 1.0 equiv) in Acetonitrile (10 mL) was added Fluolead (3.0 mmol, 1.0 equiv) at rt. The reaction mixture was stirred at rt for 2 hours, and then quenched with water. The resulting mixture was extracted with DCM (20 mL × 2). The combined organic layers were washed with brine, dried over anhydrous Na_2SO_4, filtered and concentrated under reduced pressure. The crude mixture was purified by column chromatography (*n*-Hexane) to afford the title compound **1k** (293.0 mg, 45% yield) as a white solid.

^{1}H NMR (700 MHz, $CDCl_3$) δ: 7.89 (s, 1H), 7.70 (s, 2H), 2.48 (t, *J* = 0.9 Hz, 3H) ppm. **^{13}C NMR** (176 MHz, $CDCl_3$) δ: 156.9 (d, *J* = 343.4 Hz), 139.4, 133.4, 133.3, 133.2 (d, *J* = 3.4 Hz), 129.8 (d, *J* = 3.4 Hz), 123.9 (d, *J* = 61.5 Hz), 22.9 ppm. **^{19}F NMR** (282 MHz, $CDCl_3$) δ: 17.95 (s, 1F) ppm. **HRMS** (EI) calculated for

C_8H_6BrFO $[M]^+$: 215.9586 found 215.9592. **IR** (KBr): 1812, 1596, 1469, 1401, 1186, 898, 876, 838, 747 cm^{-1}. **m.p.**: 49.5-50.2°C.

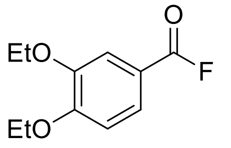

3,4-diethoxybenzoyl fluoride (1l).

To a solution of the corresponding carboxylic acid (3.0 mmol, 1.0 equiv) in Acetonitrile (10 mL) was added Fluolead (3.0 mmol, 1.0 equiv) at rt. The reaction mixture was stirred at rt for 2 hours, and then quenched with water. The resulting mixture was extracted with DCM (20 mL × 2). The combined organic layers were washed with brine, dried over anhydrous Na_2SO_4, filtered and concentrated under reduced pressure. The crude mixture was purified by column chromatography (*n*-Hexane/Et_2O = 5/1) to afford the title compound **1l** (496.2 mg, 78% yield) as a white solid.

^{1}H NMR (300 MHz, $CDCl_3$) δ: 7.76 – 7.60 (m, 1H), 7.48 (s, 1H), 6.95 – 6.19 (m, *J* = 8.6, 4.0 Hz, 1H), 4.22 – 4.13 (m, 4H), 1.55 – 1.46 (m, 6H) ppm. **^{13}C NMR** (176 MHz, $CDCl_3$) δ: 157.4 (d, *J* = 339.4 Hz), 154.8, 148.6, 126.1 (d, *J* = 2.9 Hz), 116.5 (d, *J* = 61.6 Hz), 114.8 (d, *J* = 5.5 Hz), 111.7, 64.70, 64.66, 14.6, 14.5 ppm. **^{19}F NMR** (282 MHz, $CDCl_3$) δ: 14.99 (s, 1F) ppm. **HRMS** (EI) calculated for $C_{11}H_{13}FO_3$ $[M]^+$: 212.0849 found 212.0859. **IR** (KBr): 1781, 1597, 1521, 1397, 1279,1198, 1144, 1037, 943 cm^{-1}. **m.p.**: 55.3-56.0 °C.

6. References

1. Zhao, S.; Guo, Y.; Han, E.J.; Luo, J., Liu; H.M.; Liu, C.;Xie, W.; Zhang, W.; Wang, M. *Org. Chem. Front.*, **2018**, *5*, 1143–1147.

2. Mizuta, S.; Stenhagen, I.S.; O'Duill, M.; Wolstenhulme, J.; Kirjavainen, A.K.; Forsback, S.J.; Tredwell, M.; Sandford, G.; Moore, P.R.; Huiban, M.; Luthra, S.K. *Org. Lett.,* **2013***, 15,* 2648–2651.

3. Kondo, H.; Oishi, M.; Fujikawa, K.; Amii, H. *Adv. Synth. Catal.*, **2011**, *353*, 1247–1252.

4. Zhang, X.; Yang, C. *Adv. Synth. Catal.*, **2015**, *357*, 2721–2727.

5. Kameo, H.; Yamamoto, J.; Asada, A.; Nakazawa, H.; Matsuzaka, H.; Bourissou, D. *Angew. Chem.*, **2019**, *131*, 18959–18963.

6. Trofymchuk, S.; Bugera, M.Y.; Klipkov, A.A.; Razhyk, B.; Semenov, S.; Tarasenko, K.; Starova, V.S.; Zaporozhets, O.A.; Tananaiko, O.Y.; Alekseenko, A.N.; Pustovit, Y. *J. Org. Chem.* **2020**, *85*, 3110–3124

7. Yang, Y.-D.; Iwamoto, K.; Tokunaga, E.; Shibata, N. *Chem. Commun.*, **2013**, *49*, 5510–5512.

8. Cloux, R.; Kovats, E. *Synthesis*, **1992**, *4*, 409–412.

9. Duddeck, H.; Spitzer, M.; Bolte, G. *Liebigs Ann. Chem.,* **1985**, *3*, 545–554.

10. Hafner, A; Bräse, S. *Adv. Synth. Catal.,* **2011**, *353*, 3044–3048.

11. Liang, Y.; Zhao, Z.; Shibata, N. *Commun. Chem.,* **2020**, *3*, 59.

12. Scattolin, T.; Deckers, K.; Schoenebeck, F. *Org. Lett.*, **2017**, *19*, 5740–5743.

13. Munoz, S.B.; Dang, H.; Ispizua-Rodriguez, X.; Mathew, T.; Prakash, G.S. *Org. Lett.*, **2019**, *21*, 1659–1663.

14. Han, J.; Zhou, W.; Zhang, P.C.; Wang, H.; Zhang, R.; Wu, H.H.; Zhang, J. *ACS Catal.* **2019**, *9*, 6890–6895.

7. ^{1}H NMR Spectra, ^{19}F NMR Spectra and ^{13}C NMR Spectra

4-(Trifluoromethyl)-1,1'-biphenyl (2a)

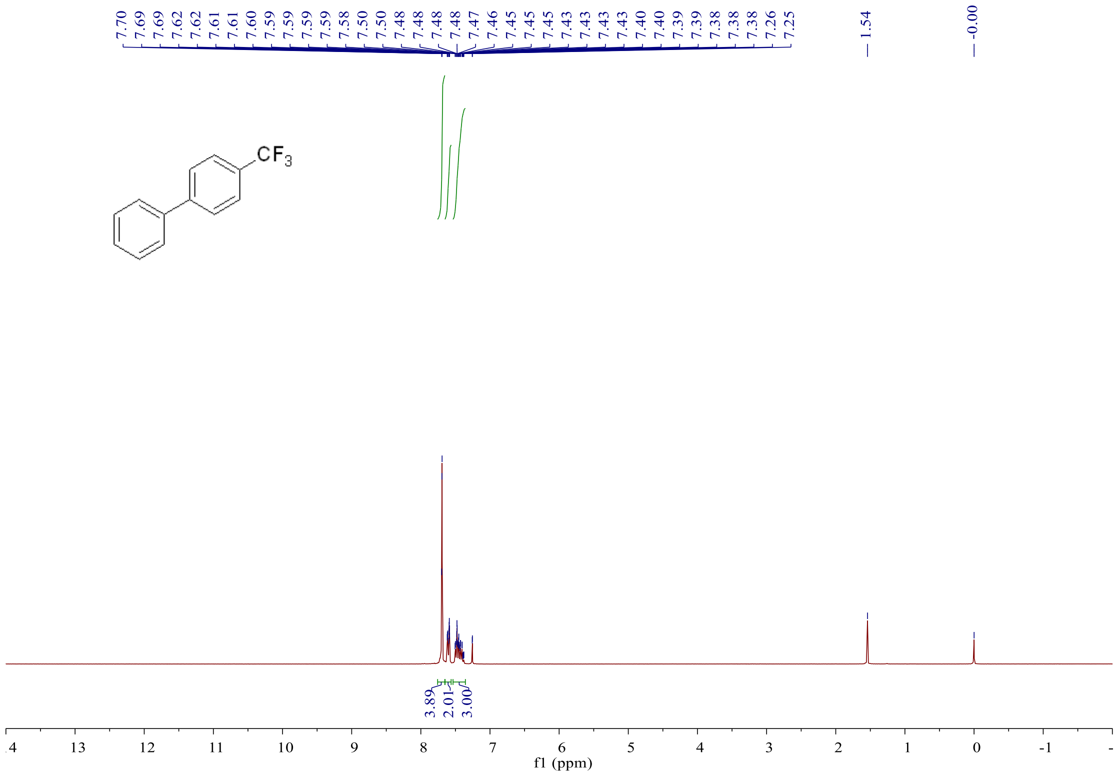

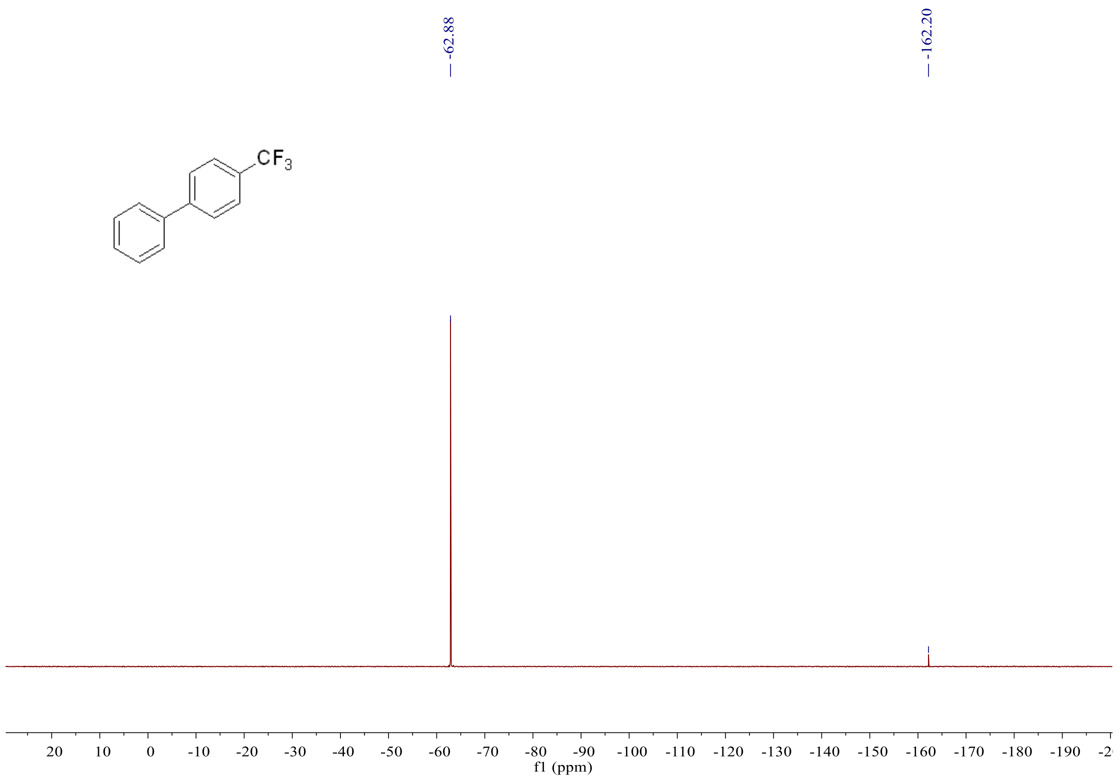

2-(Trifluoromethyl)-1,1'-biphenyl (2b).

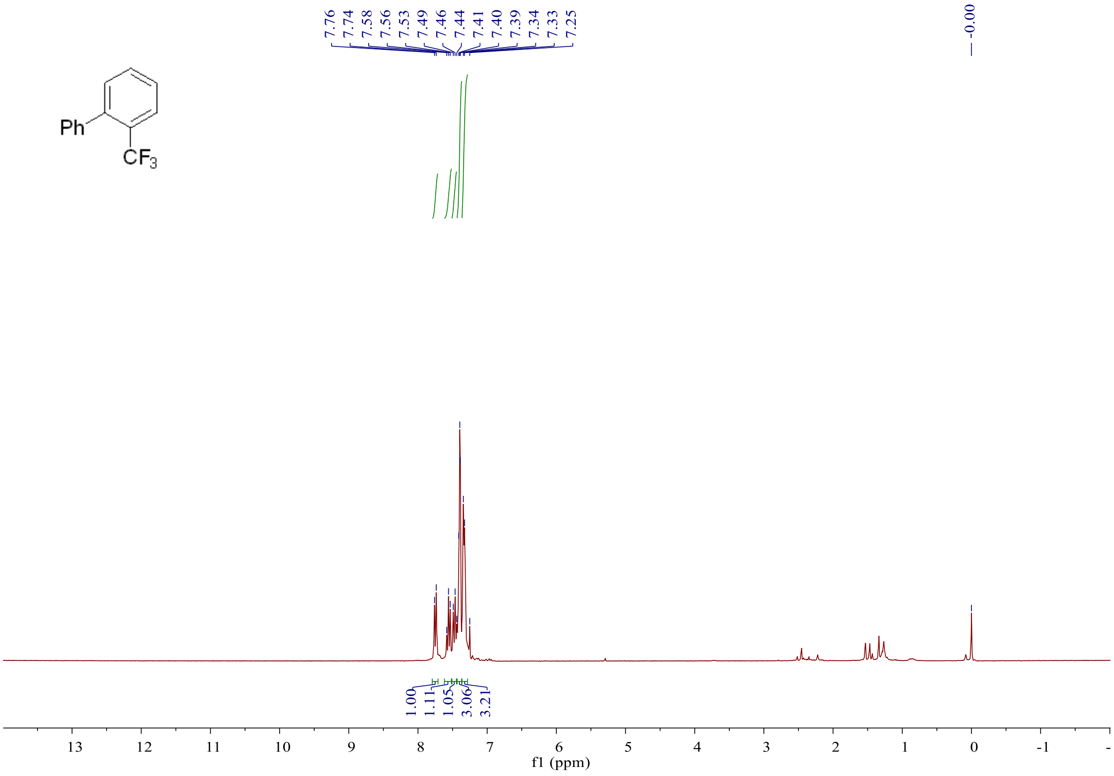

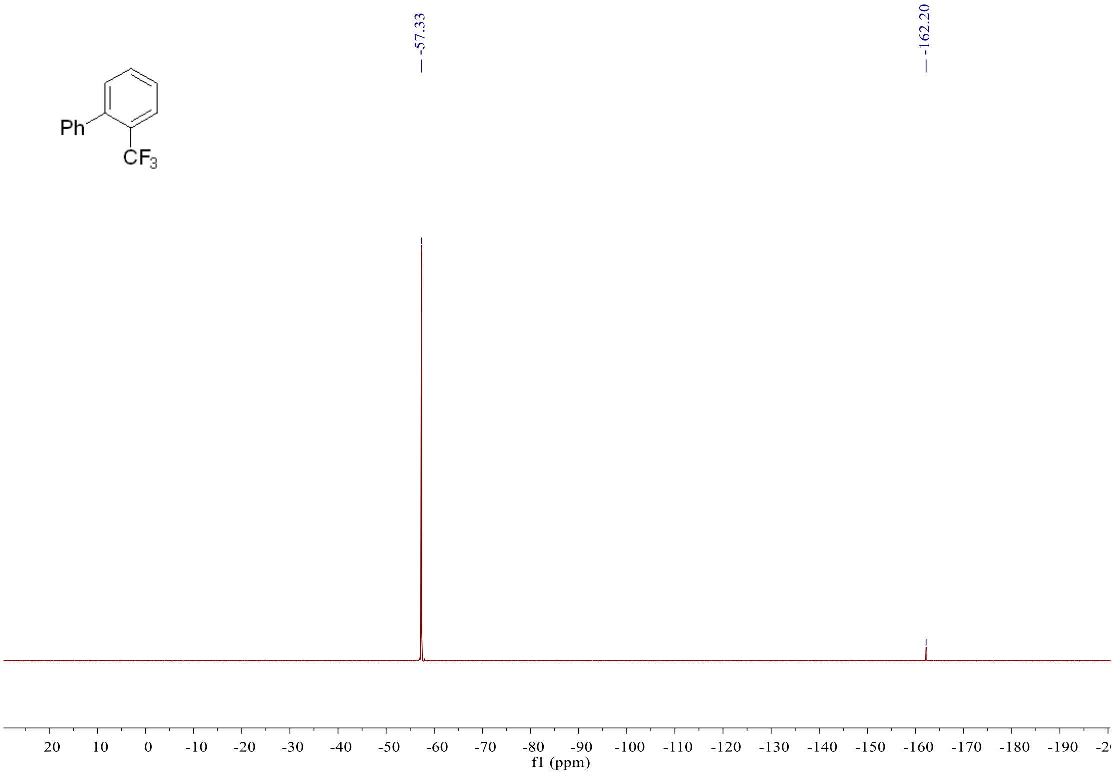

1-Bromo-4-(trifluoromethyl)benzene (2c).

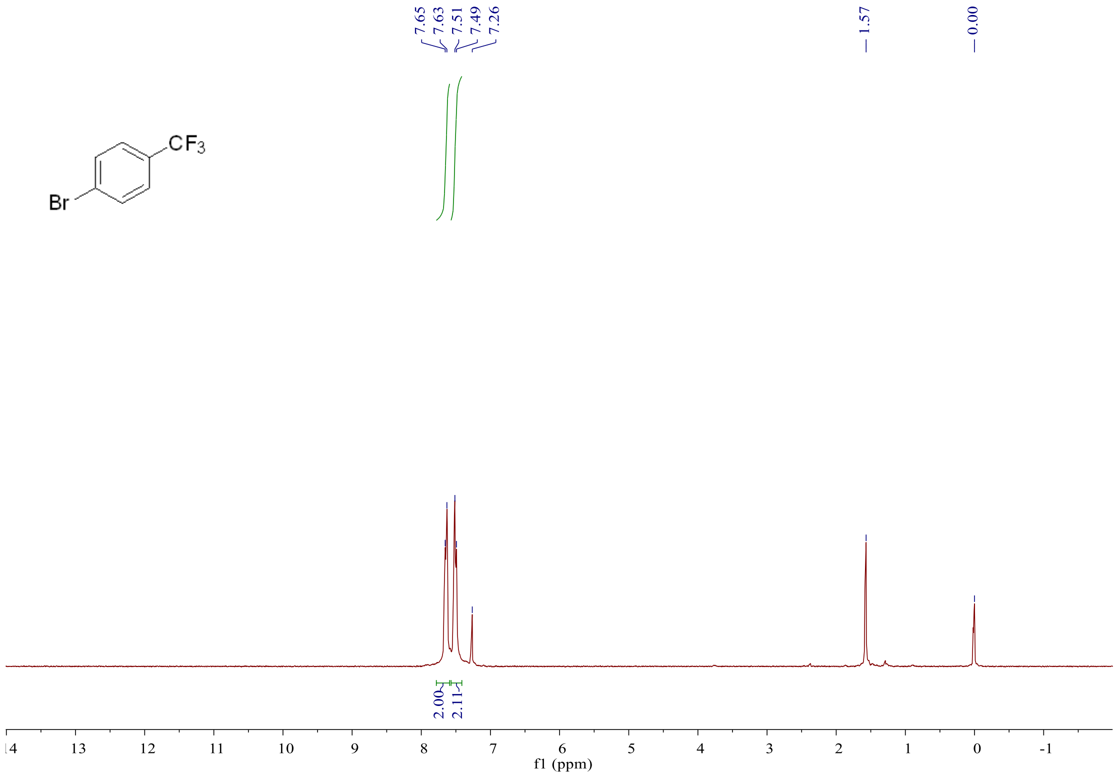

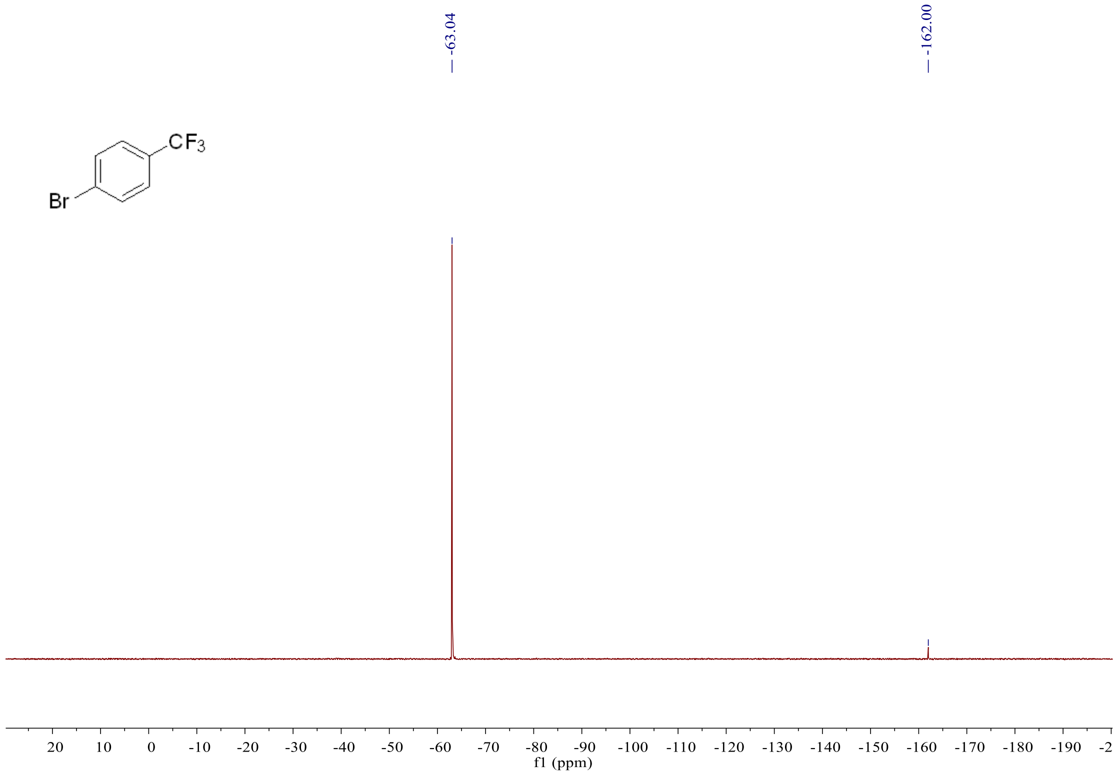

1-Butyl-4-(trifluoromethyl)benzene (2d).

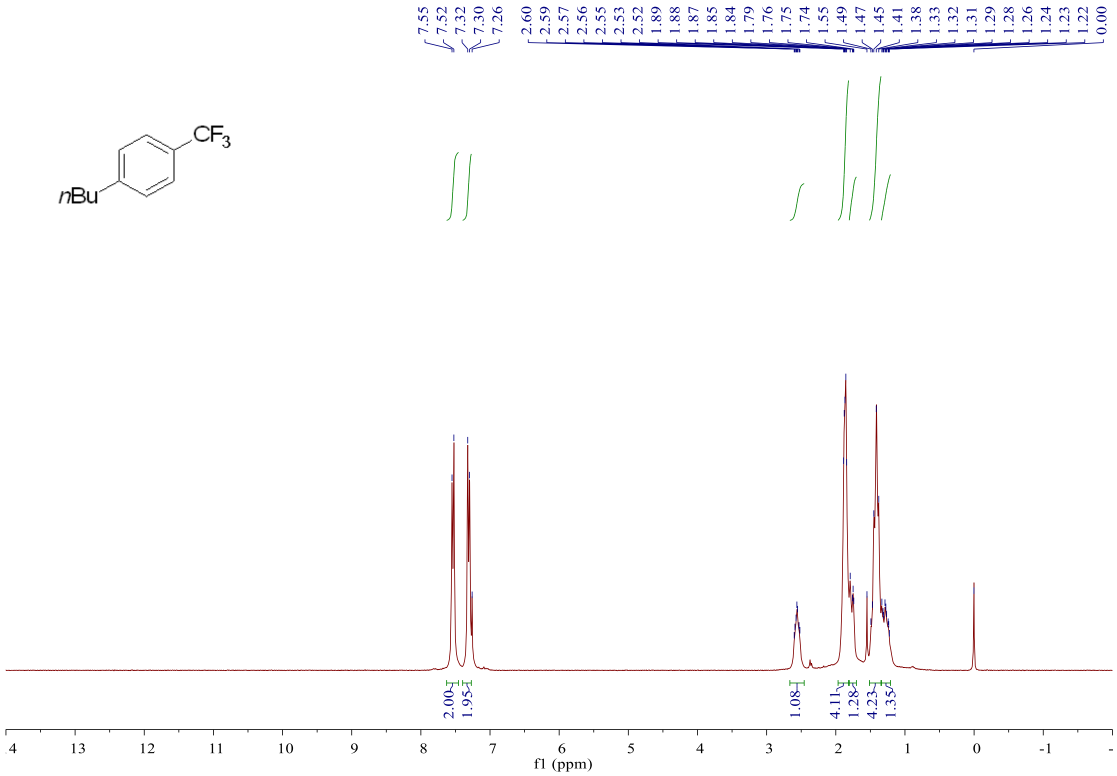

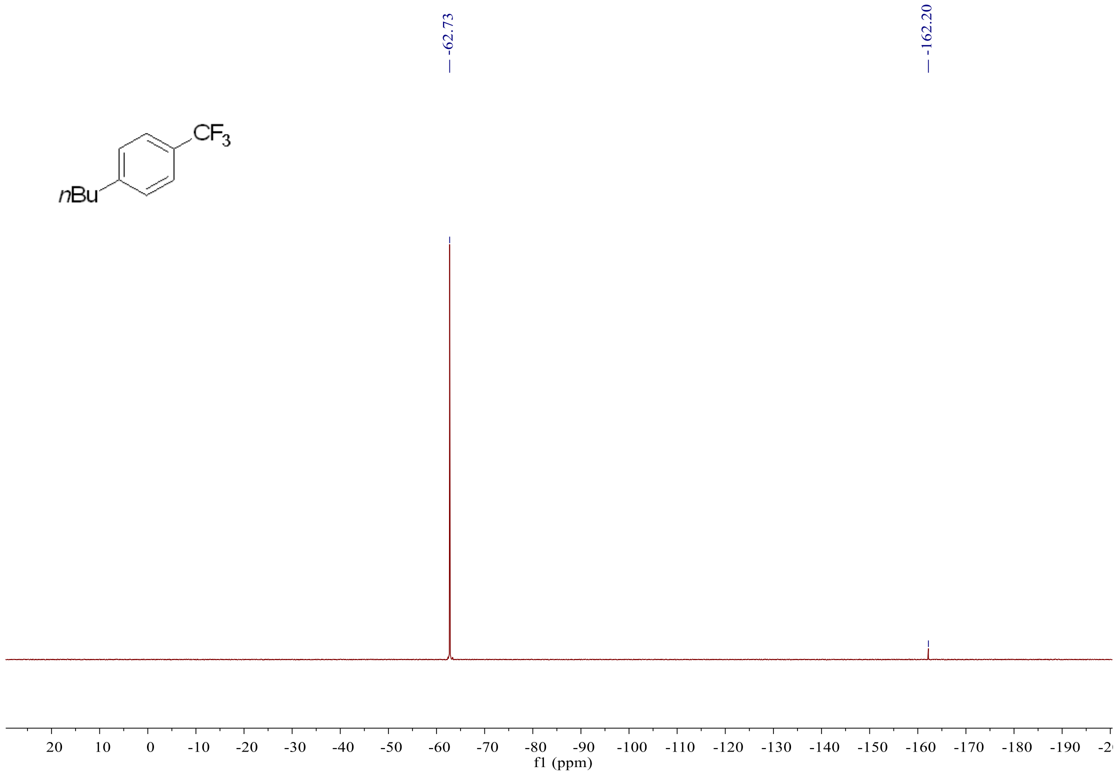

1-Cyclohexyl-4-(trifluoromethyl)benzene (2e).

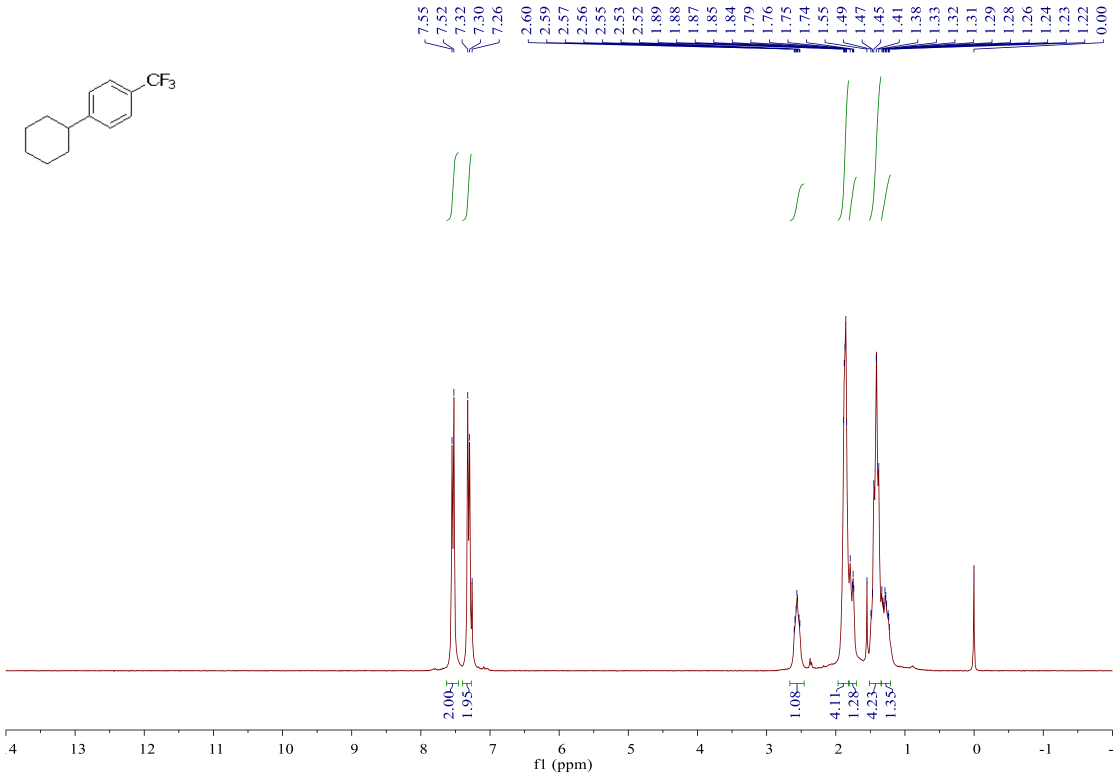

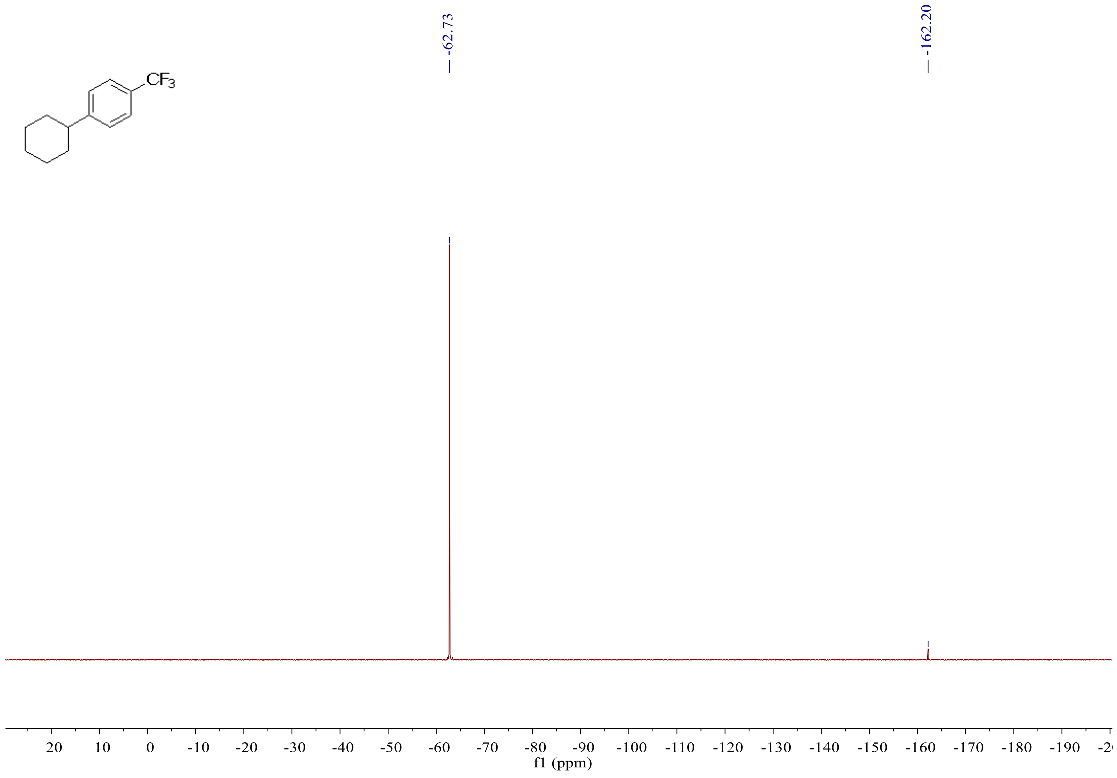

1-Methyl-4-(trifluoromethyl)benzene (2f).

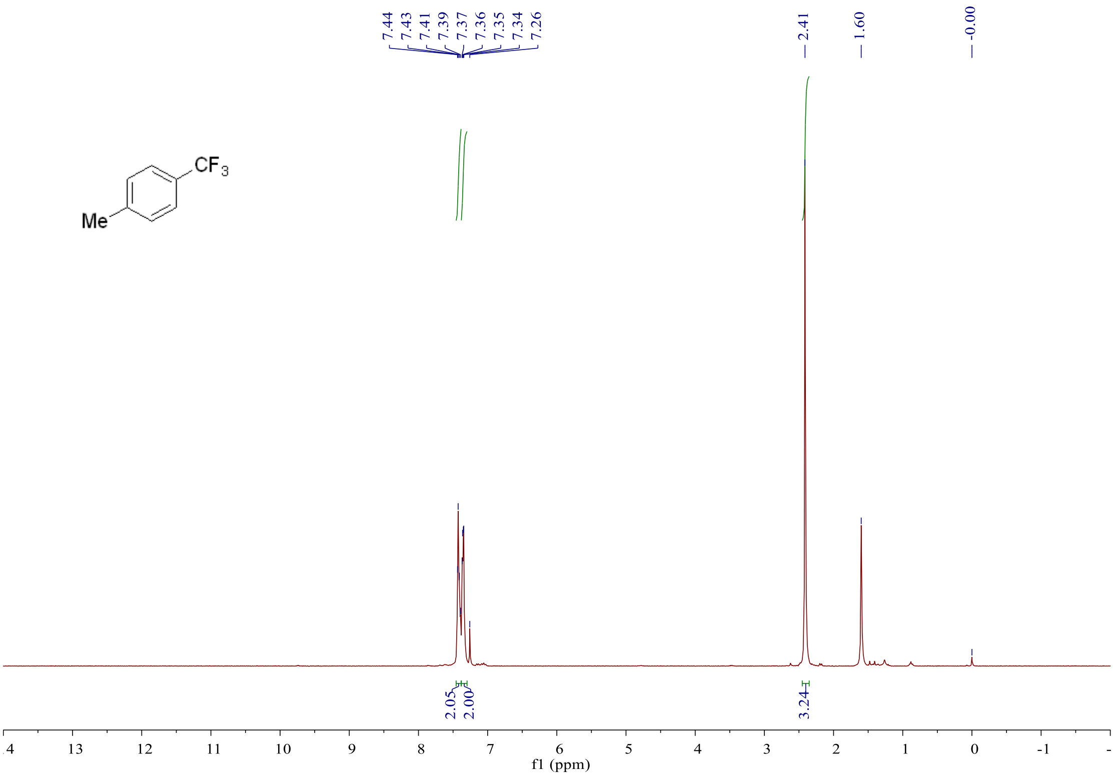

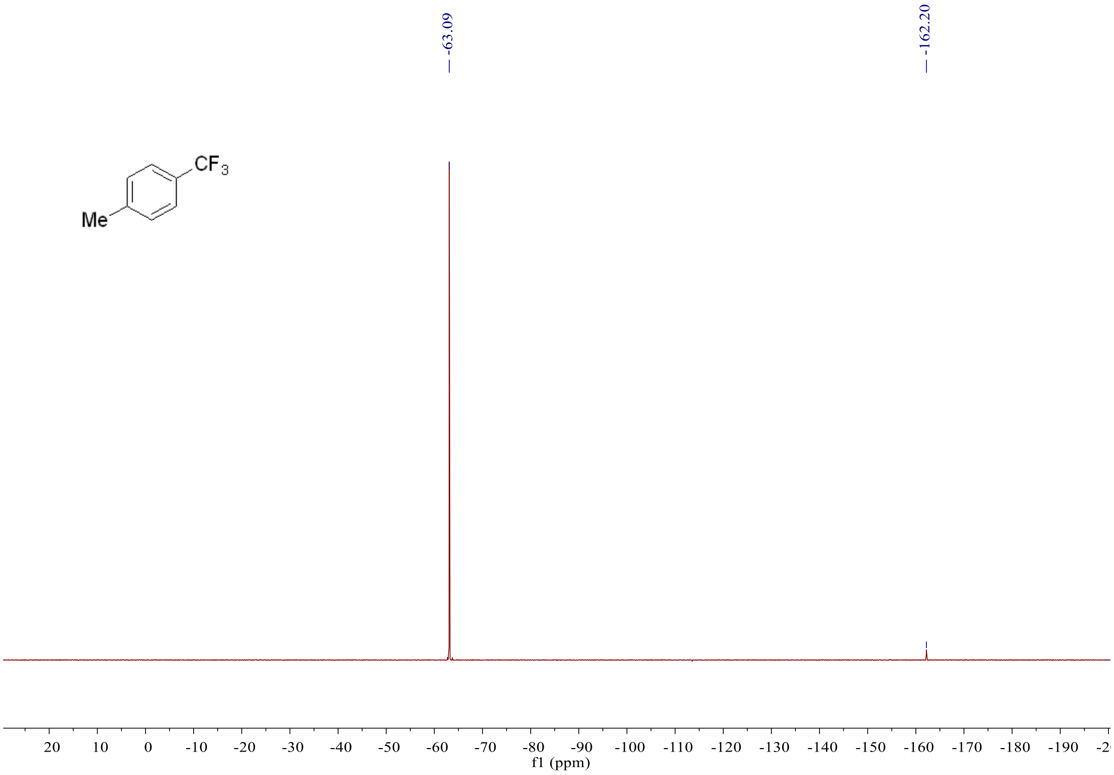

2-(Trifluoromethyl)naphthalene (2g).

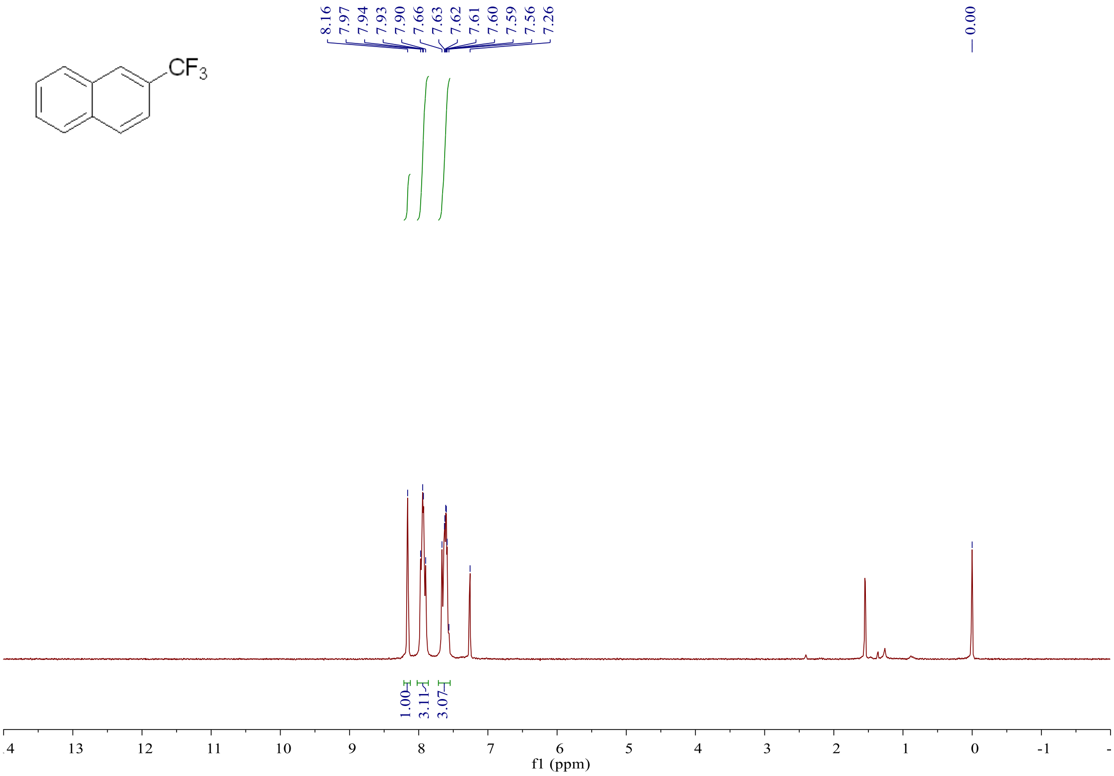

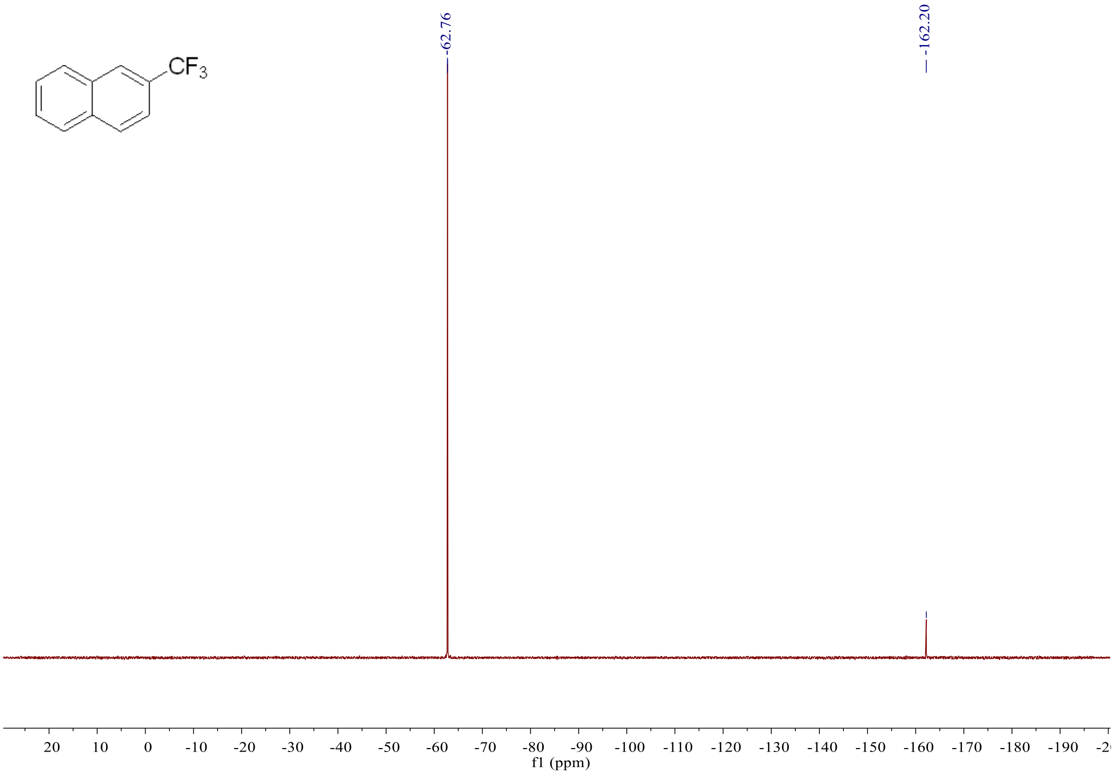

1-(Trifluoromethyl)naphthalene (2h).

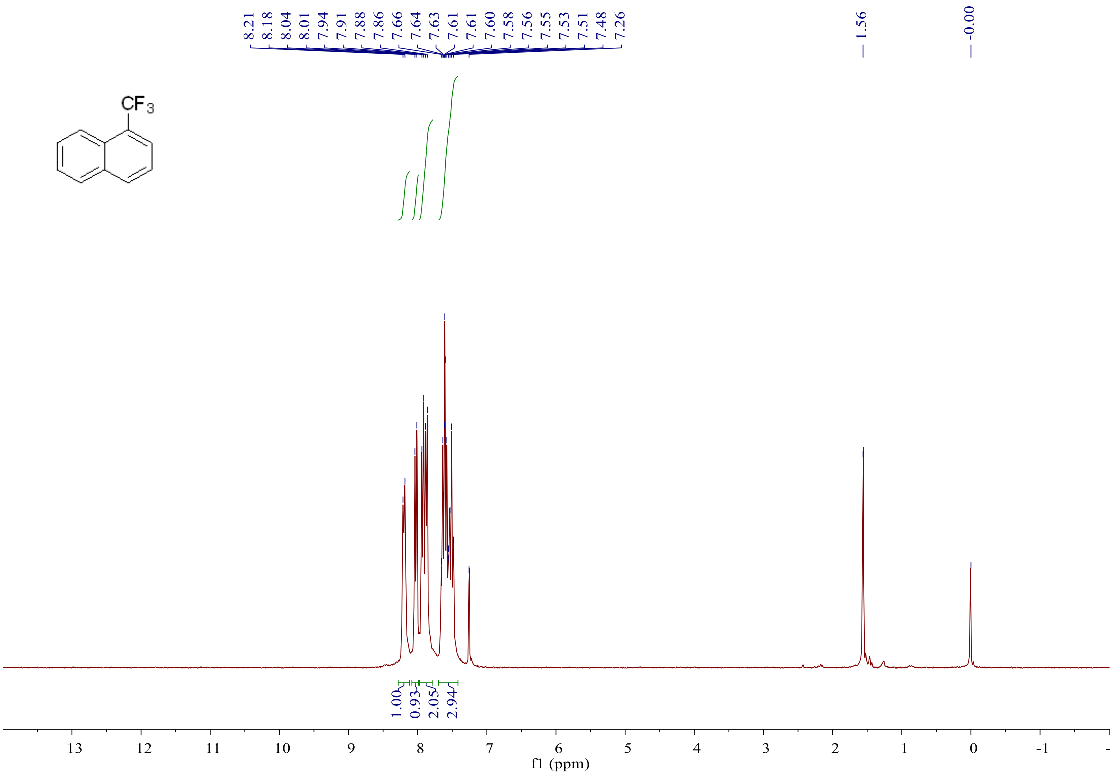

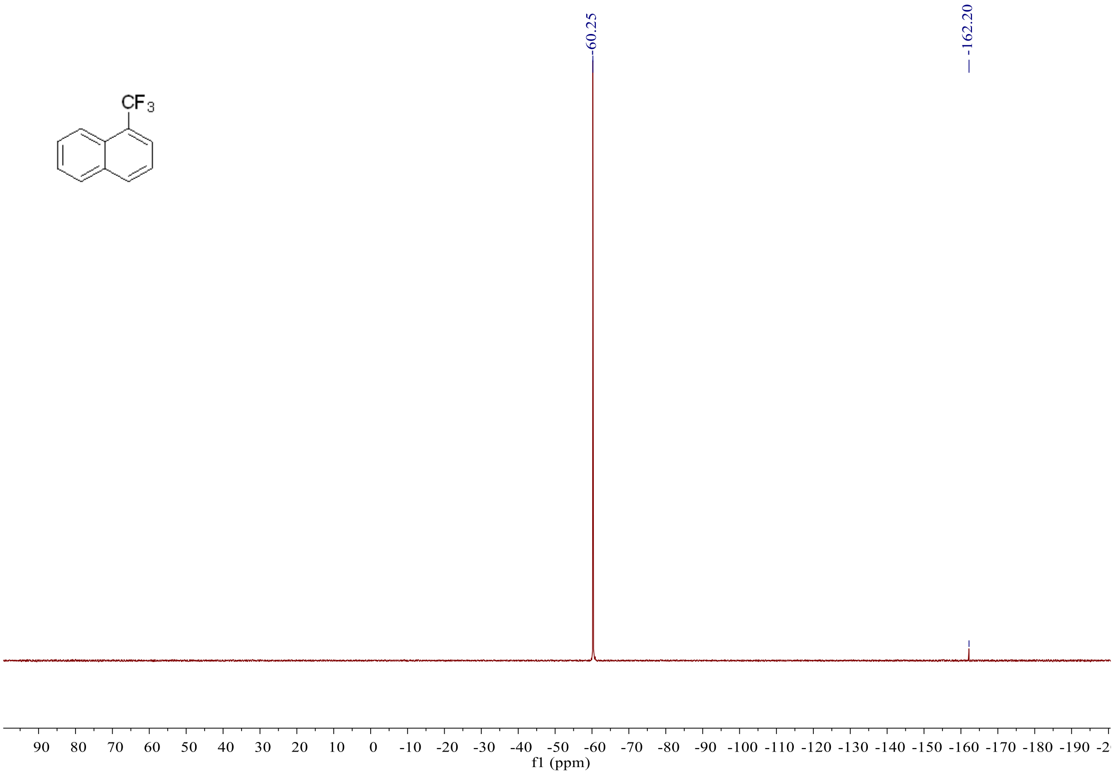

(Trifluoromethyl)benzene (2i).

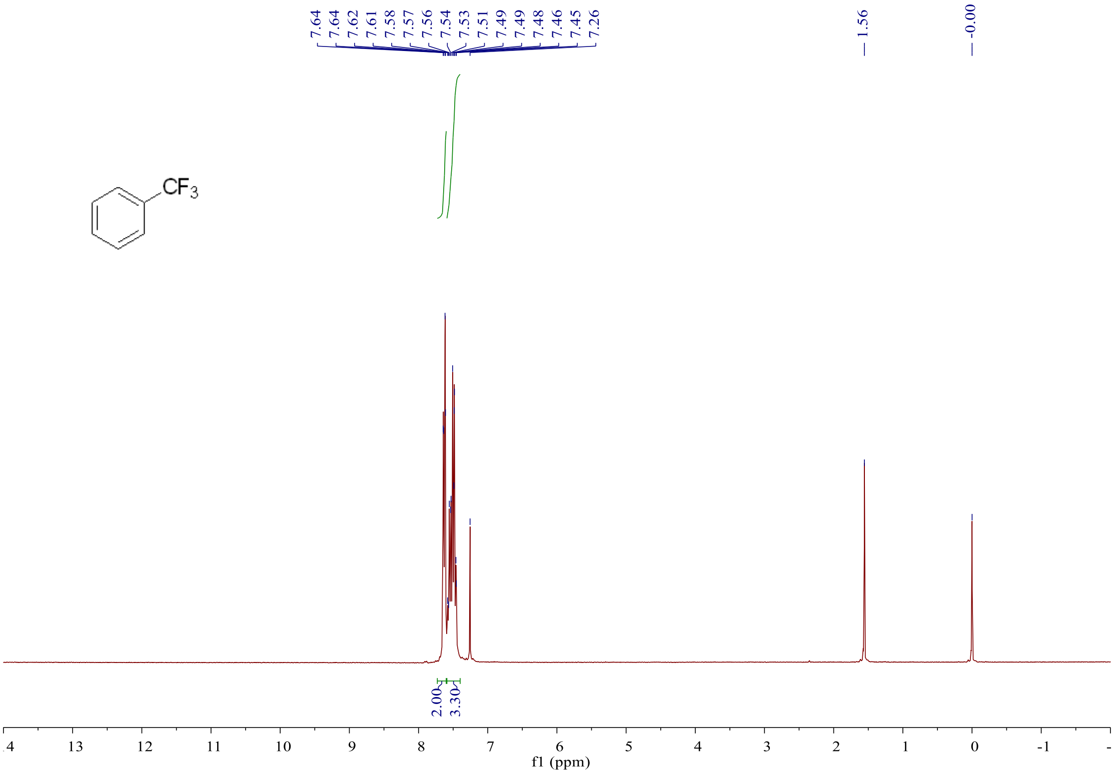

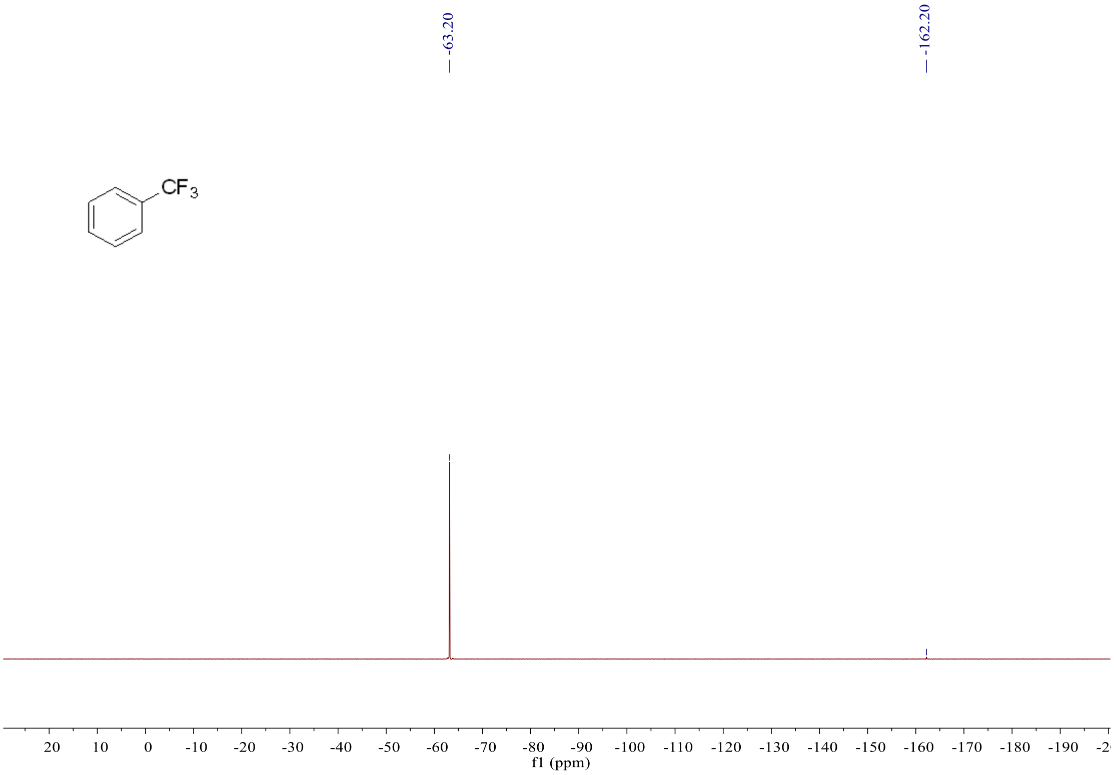

1,3-Di-*tert*-butyl-5-(trifluoromethyl)benzene (2j).

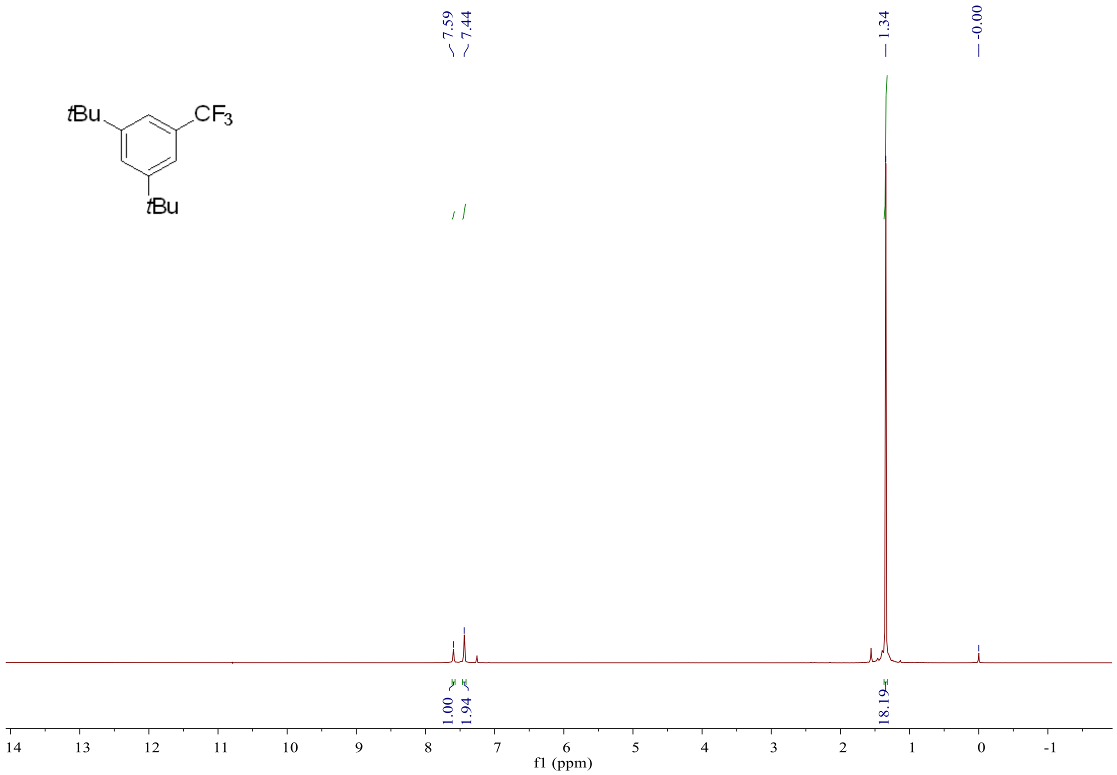

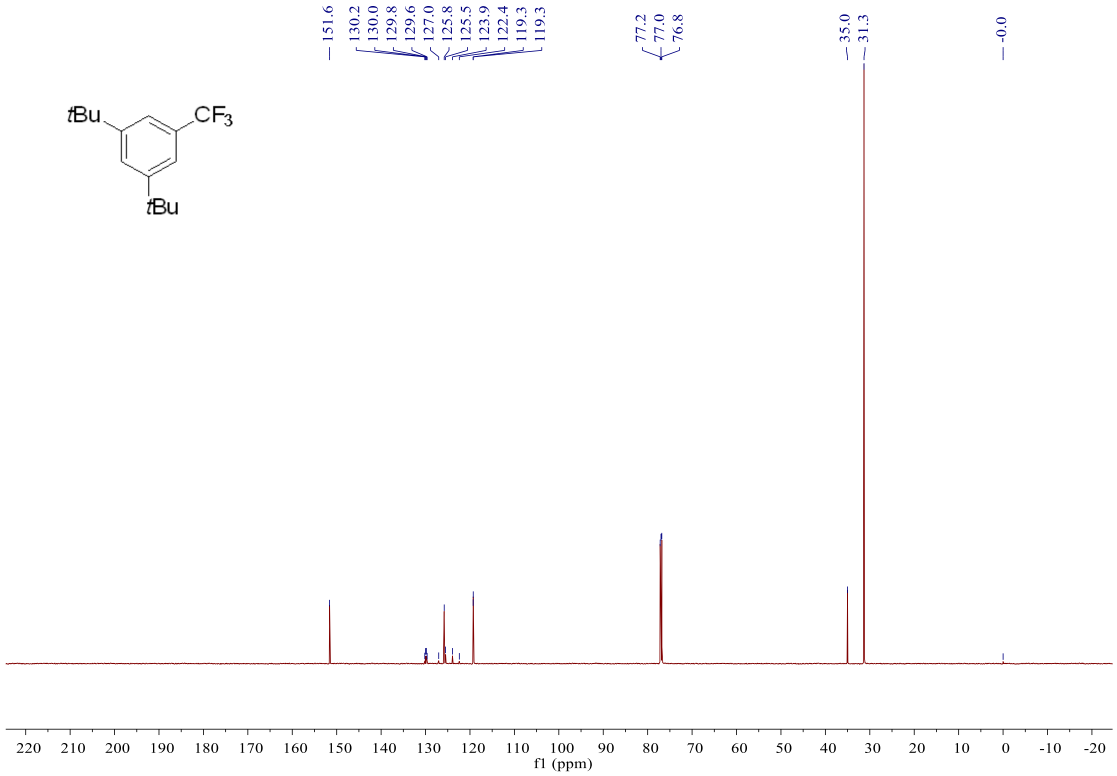

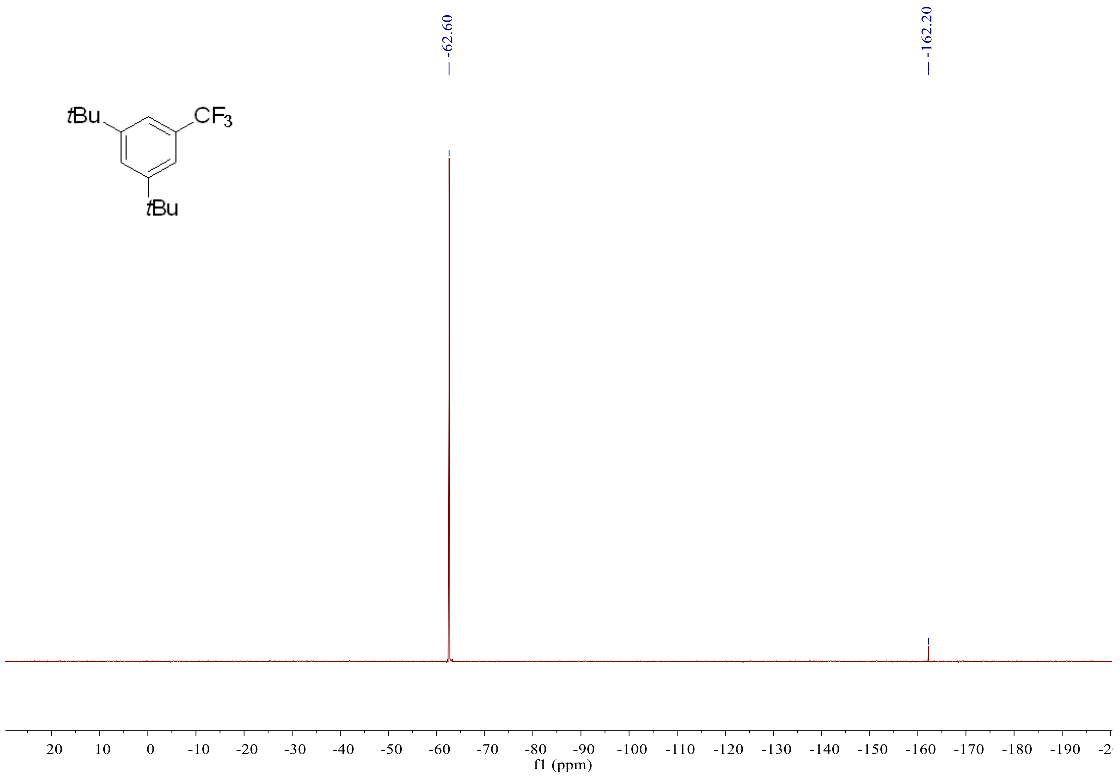

1-Bromo-2-methyl-4-(trifluoromethyl)benzene (2k).

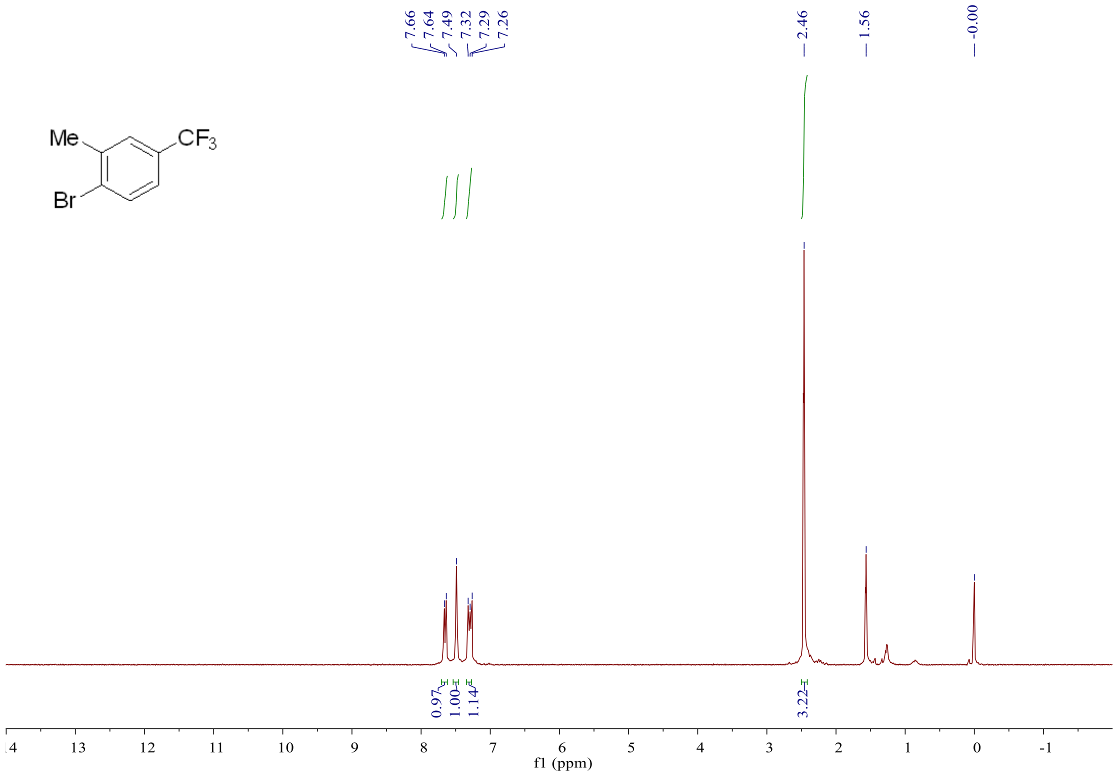

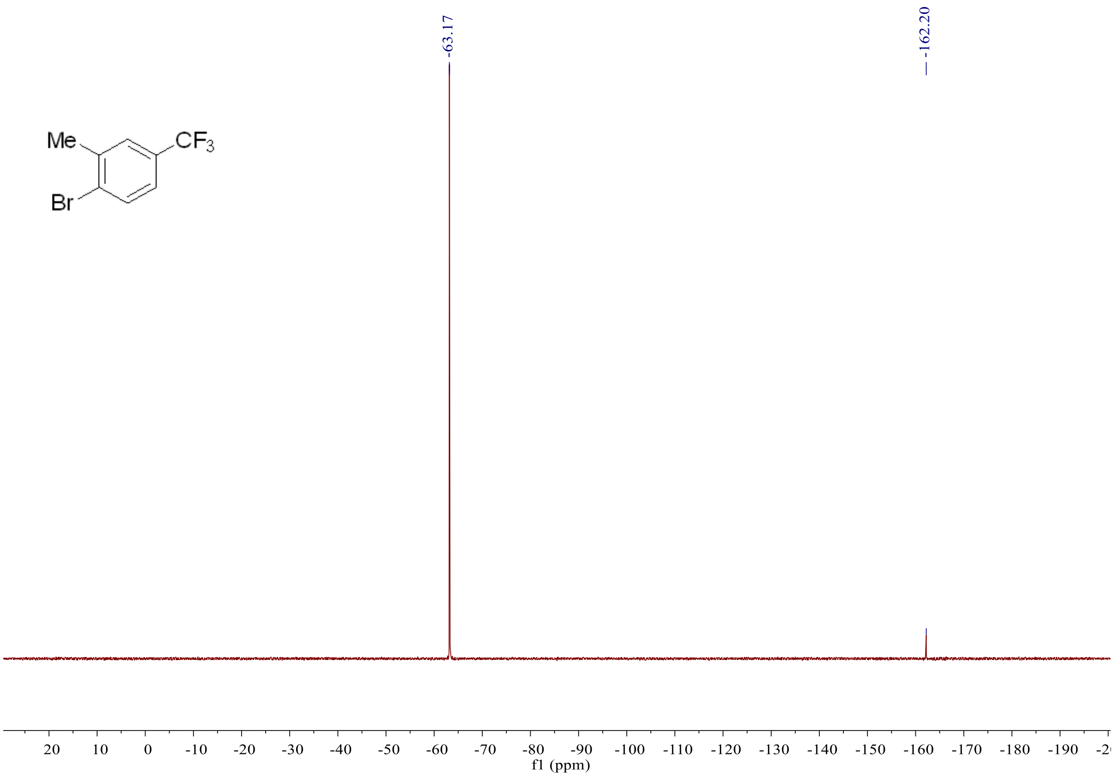

1,2-Diethoxy-4-(trifluoromethyl)benzene (2l).

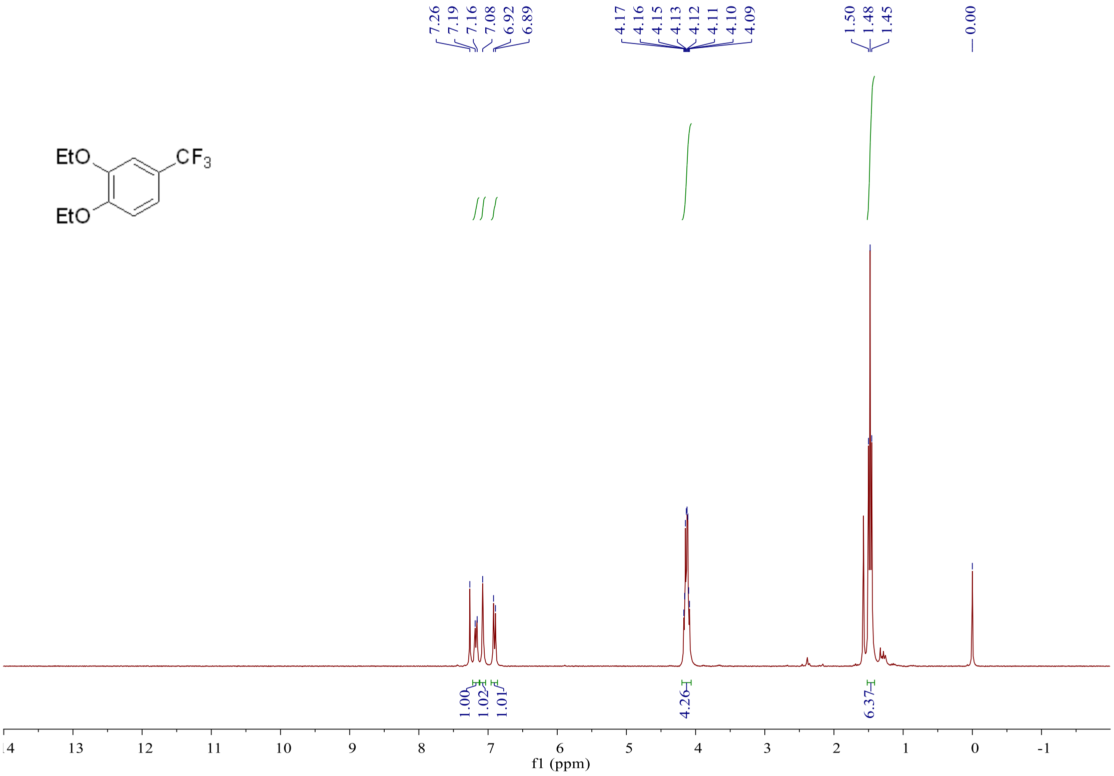

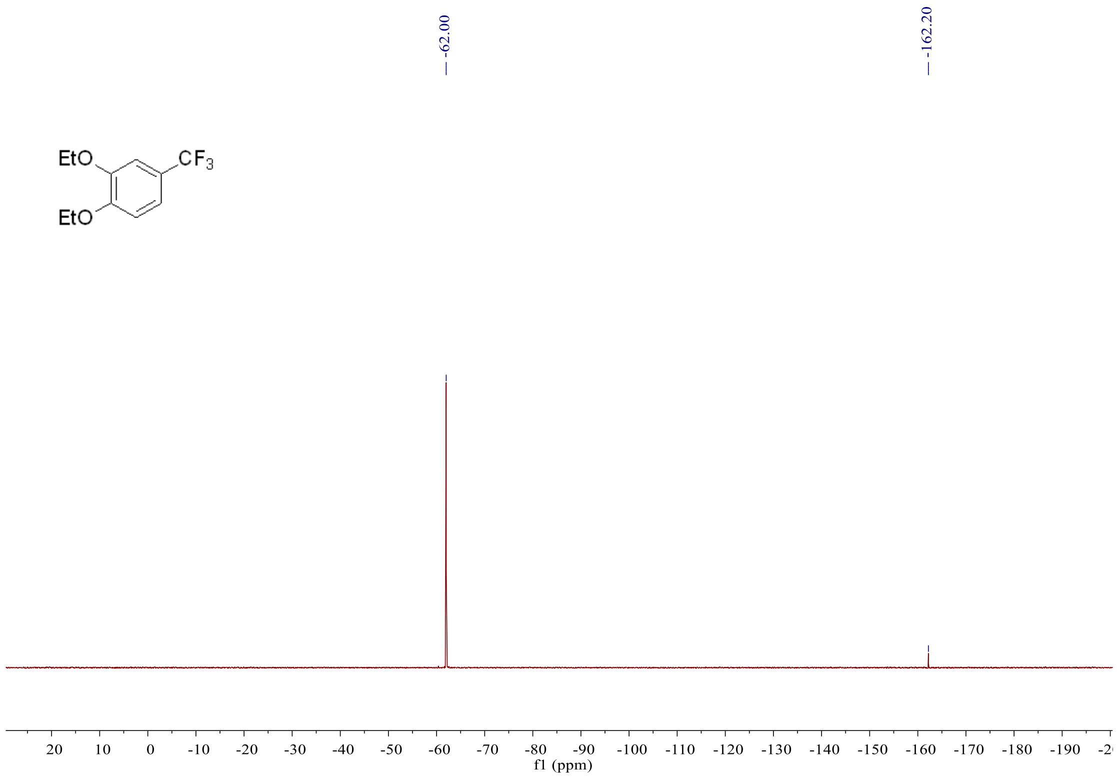

2-(Trifluoromethyl)thiophene (2m).

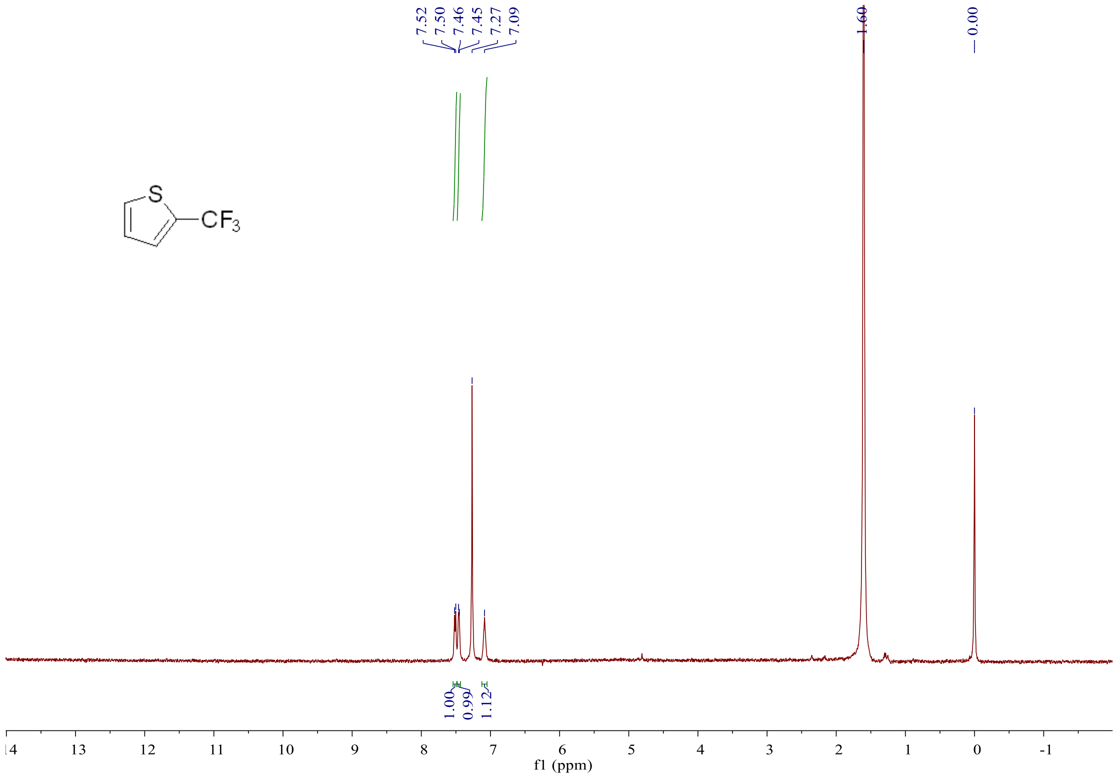

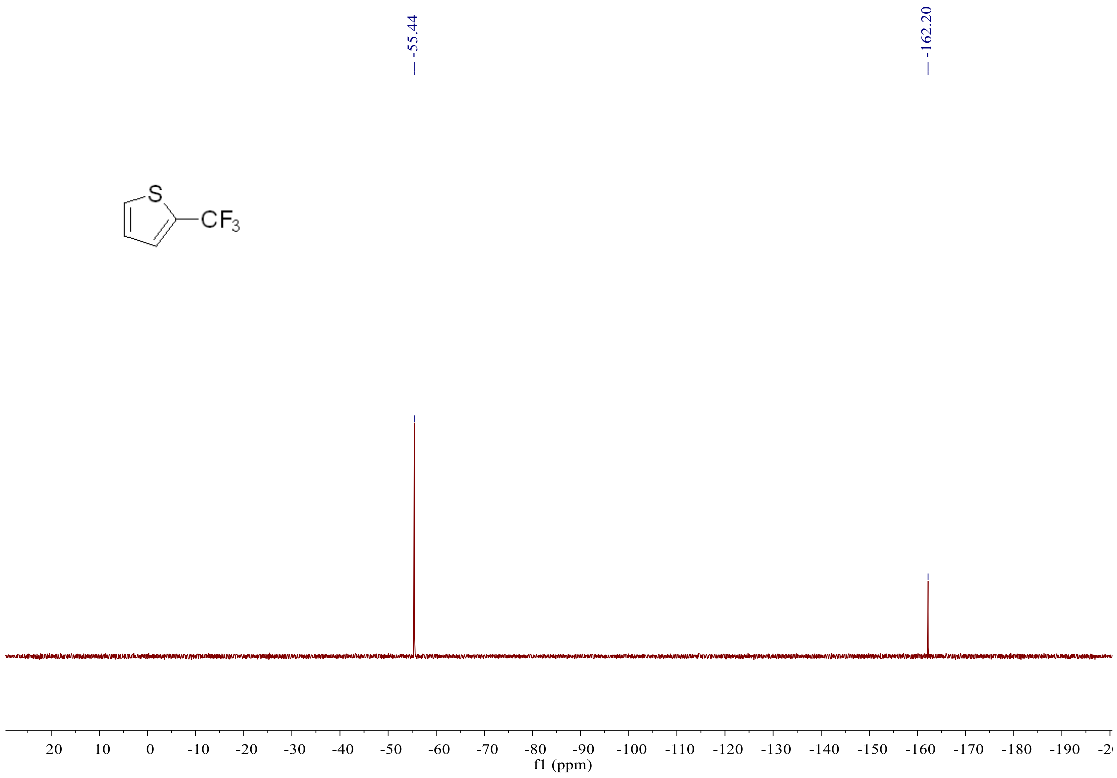

1,1,1-Trifluorododecane (2n).

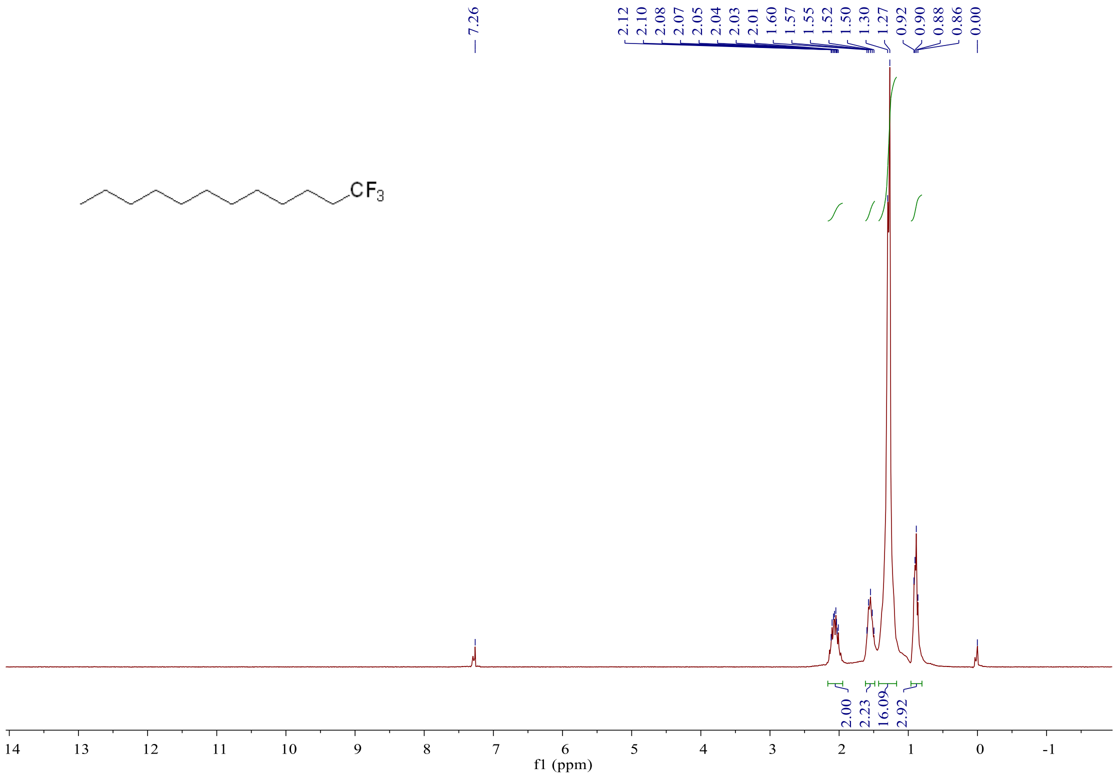

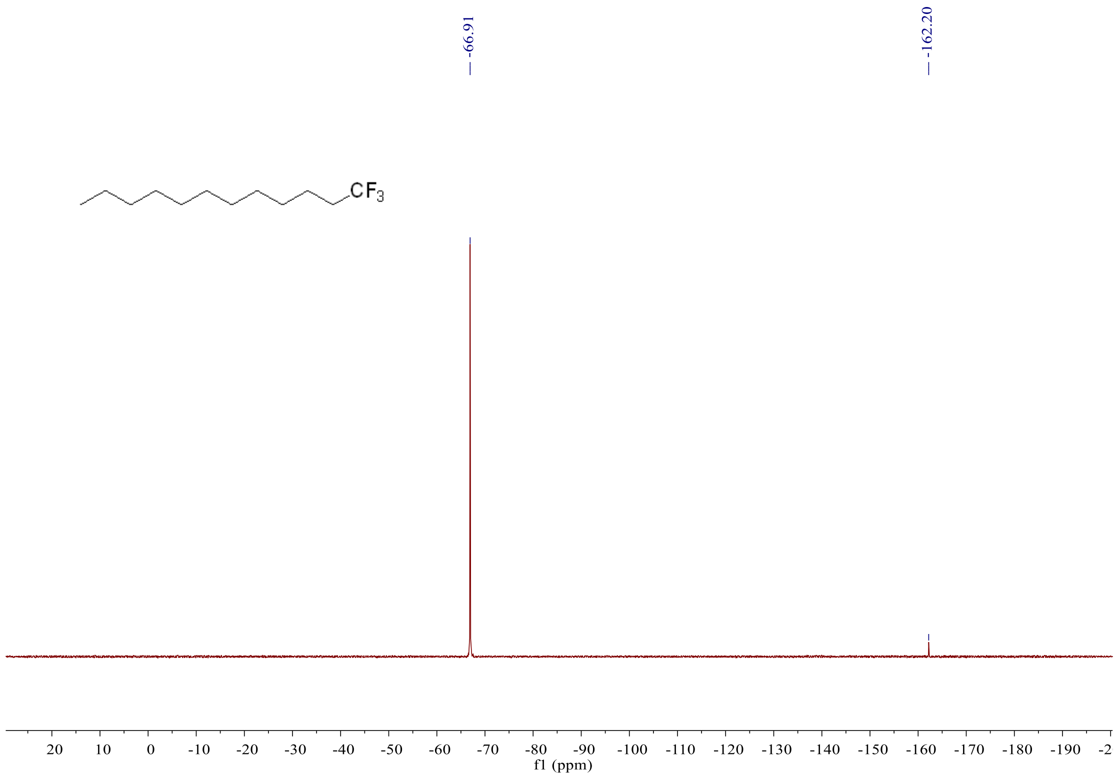

1-Trifluoromethyladamantane (2o).

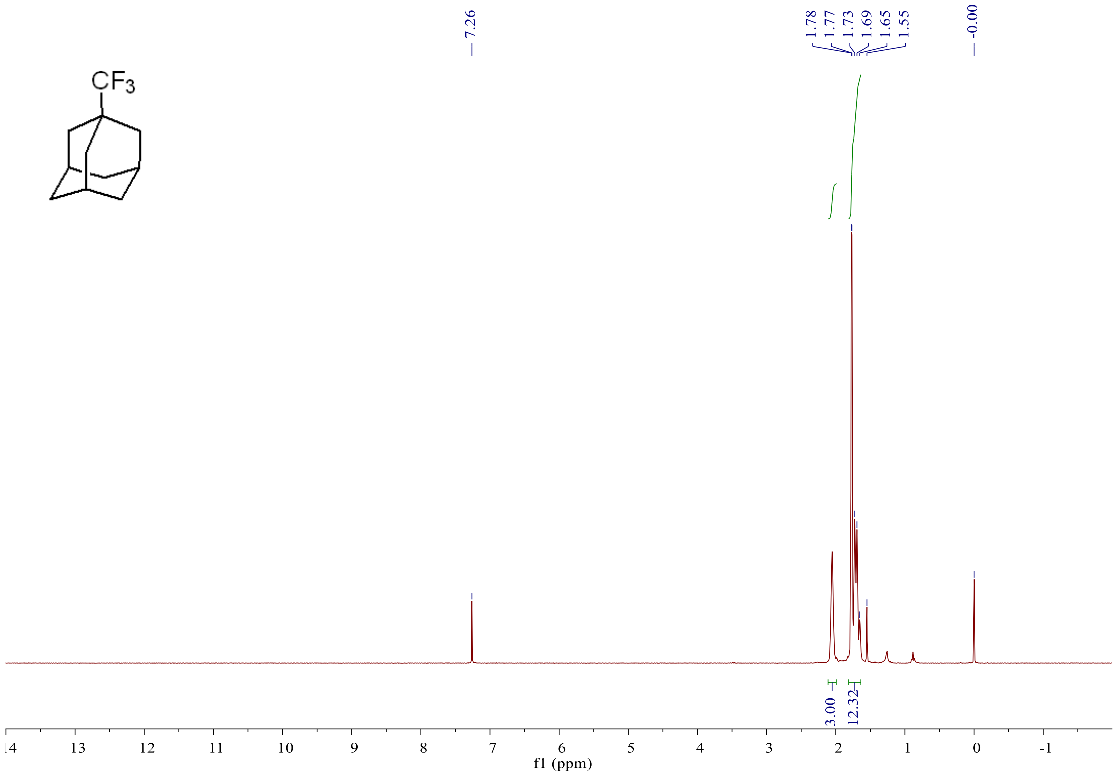

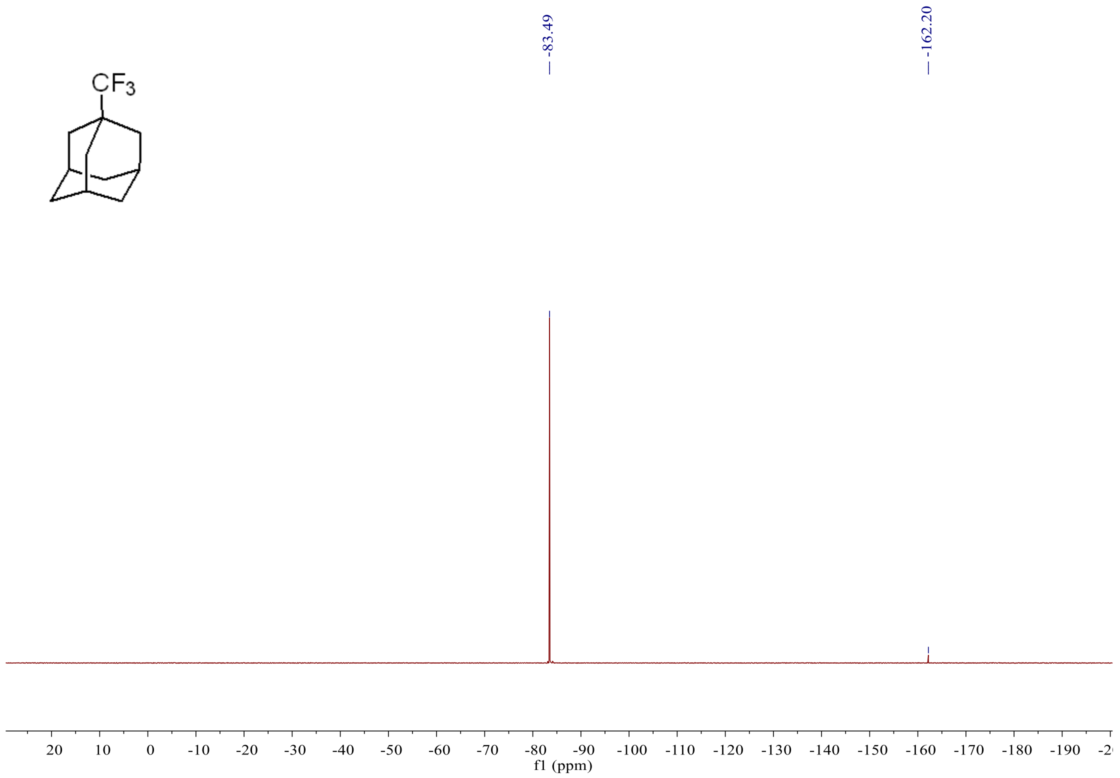

(*E*)-1-Phenyl-3,3-trifluoroprop-1-ene (2p).

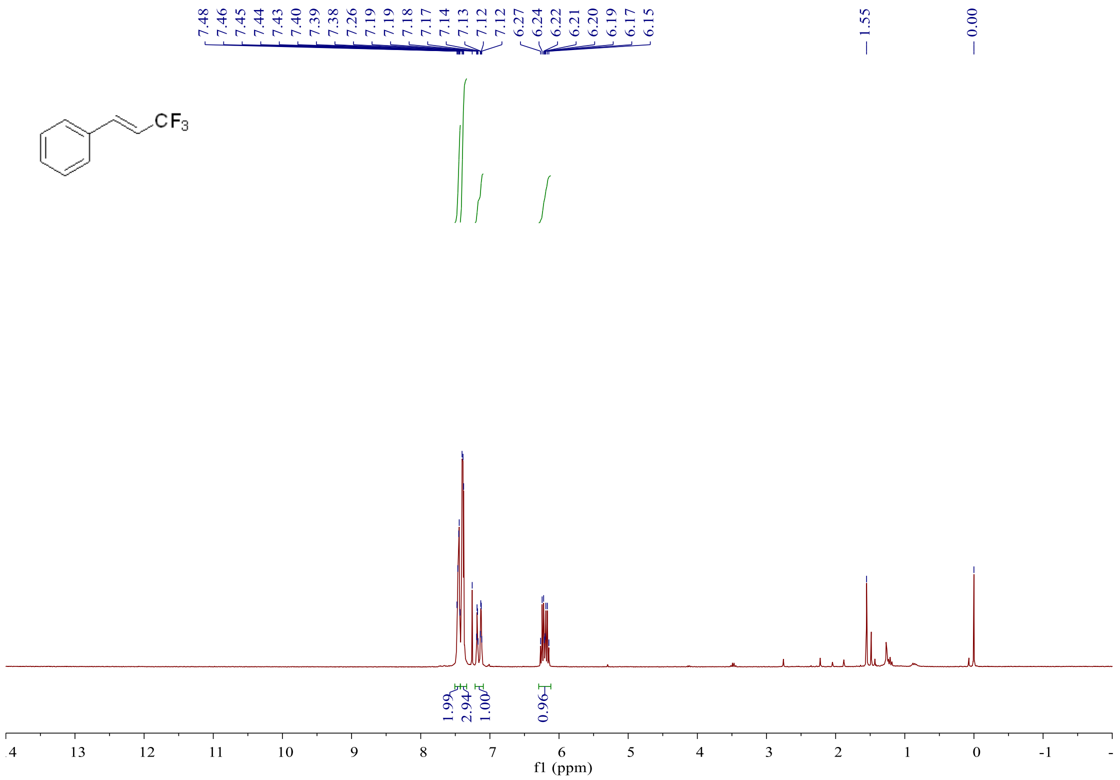

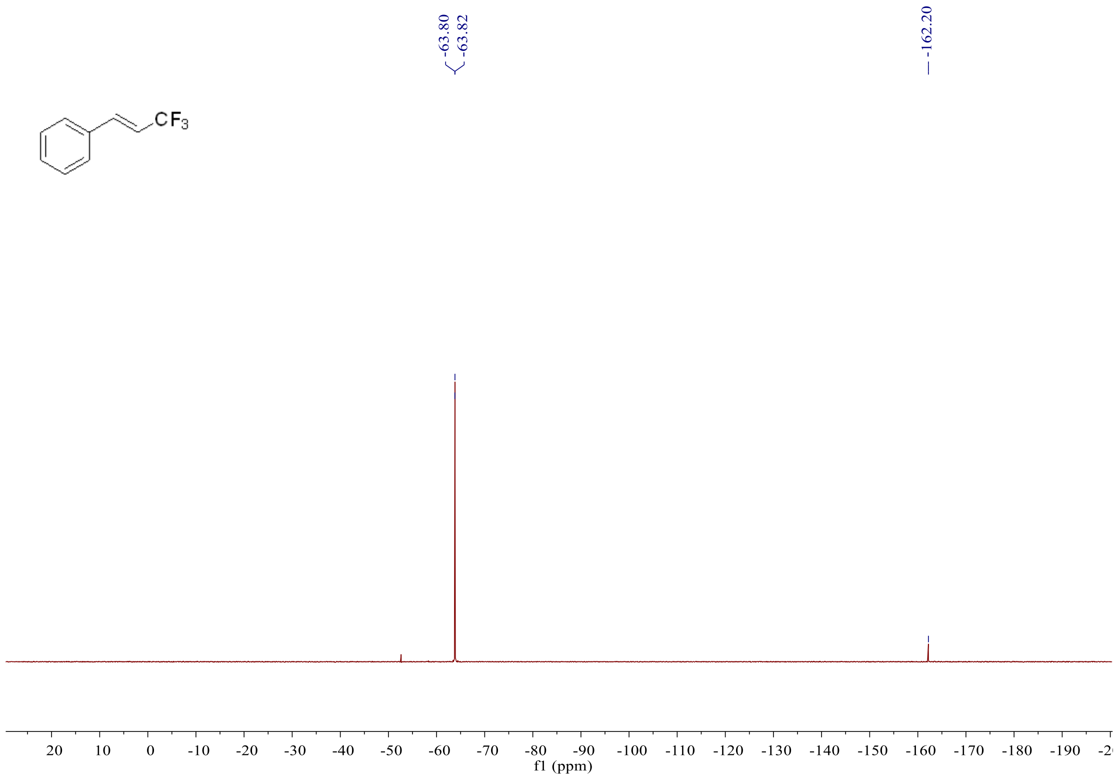

_N,N_-Dipropyl-4-(trifluoromethyl)benzenesulfonamide (2q).

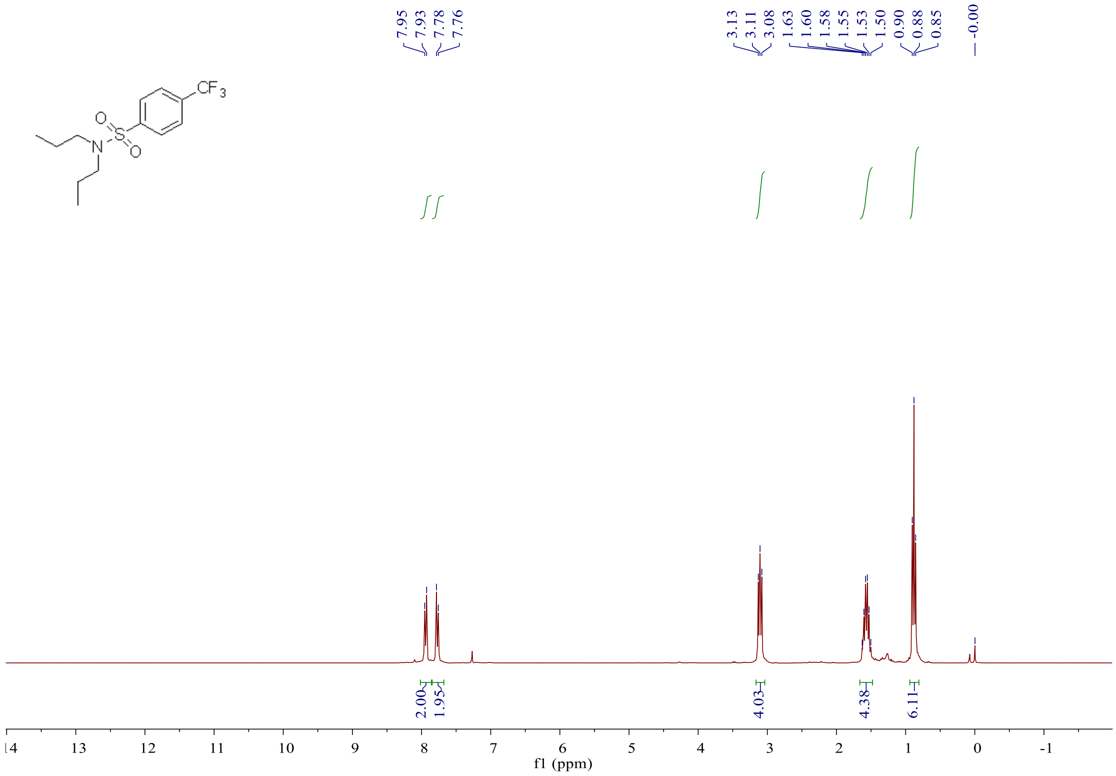

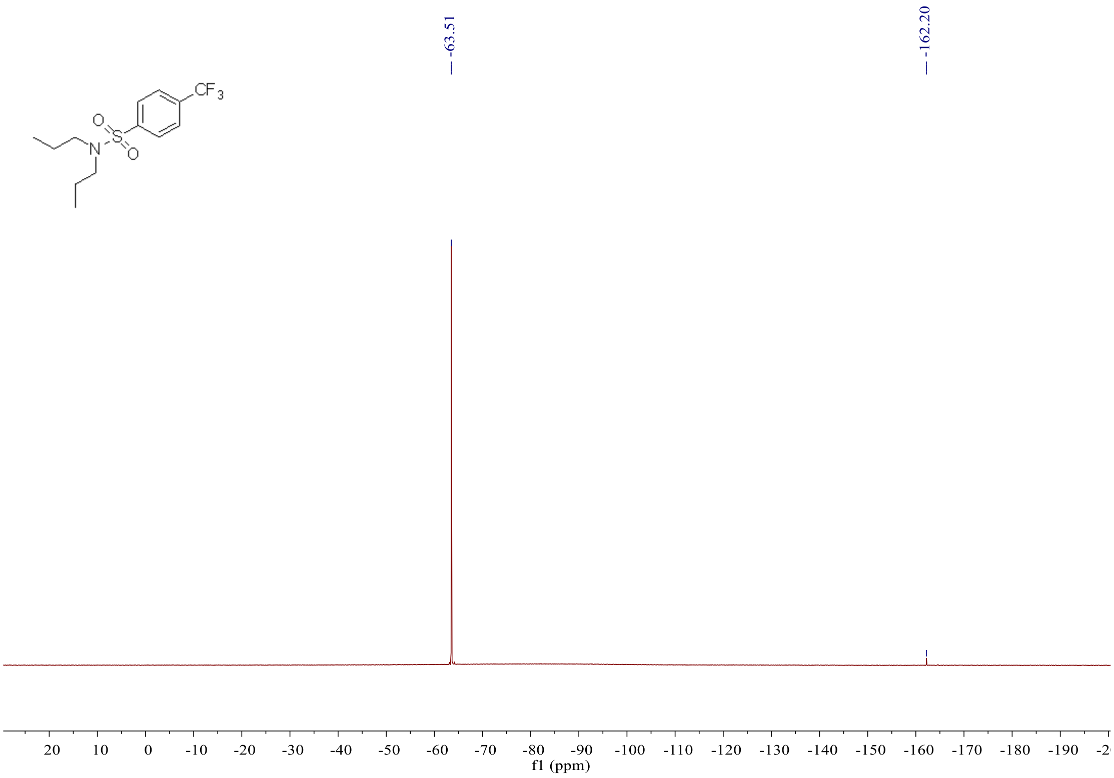

2-Isobutoxy-5-(4-methyl-5-(trifluoromethyl)thiazol-2-yl)benzonitrile (2r).

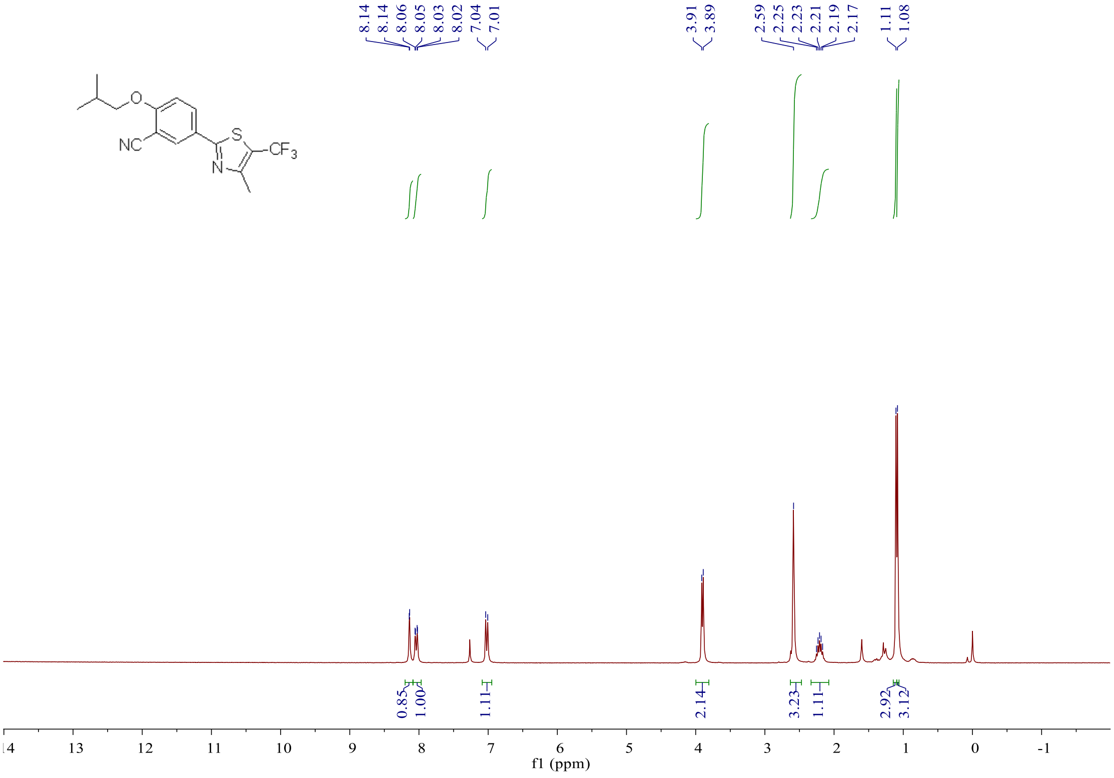

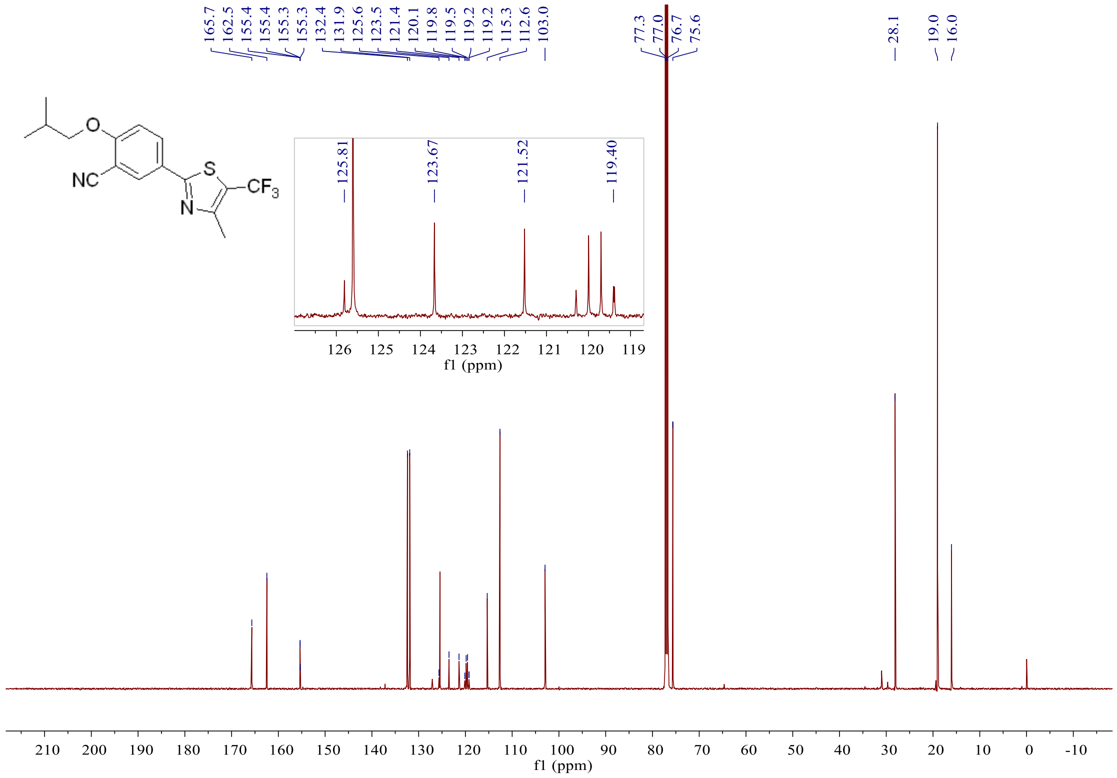
165.7
162.5
155.4
155.4
155.3
155.3
132.4
131.9
125.6
123.5
121.4
120.1
119.8
119.5
119.2
119.2
115.3
112.6
103.0
77.3
77.0
76.7
75.6
28.1
19.0
16.0
NC
O
S
N
CF3
125.81
123.67
121.52
119.40
f1 (ppm)
f1 (ppm)

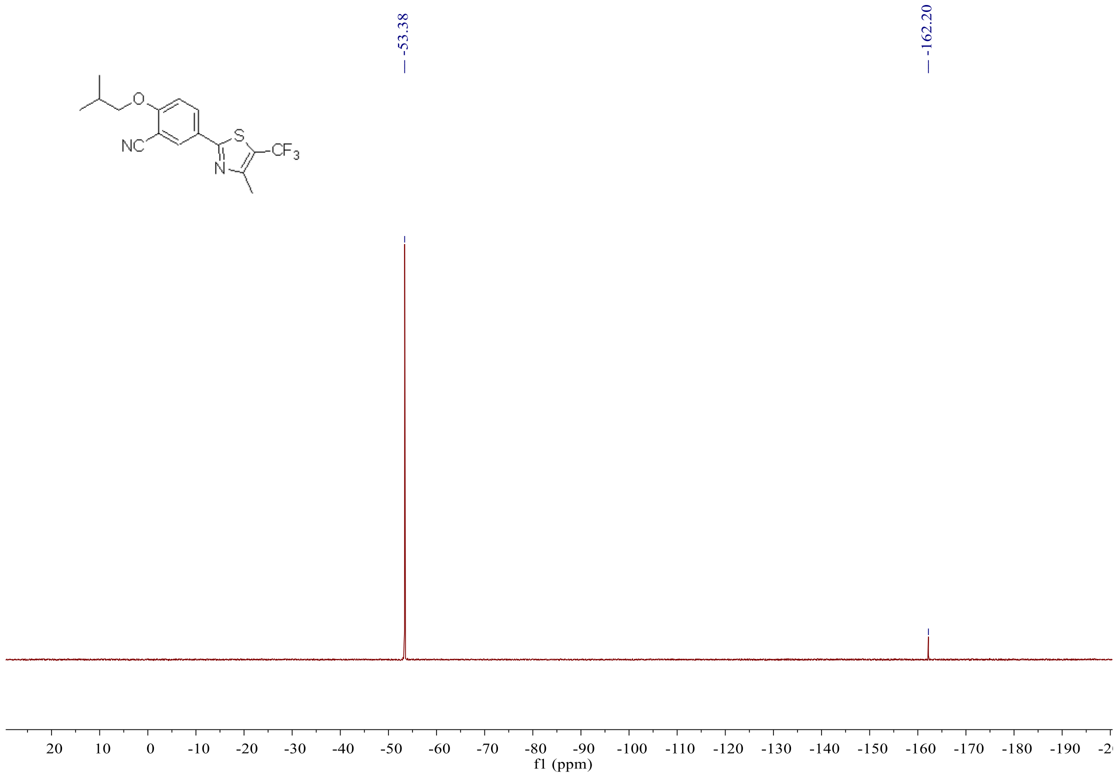
-53.38
-162.20
NC
O
S
N
CF3
f1 (ppm)

3,5-Di-*tert*-butylbenzoyl fluoride (1j).

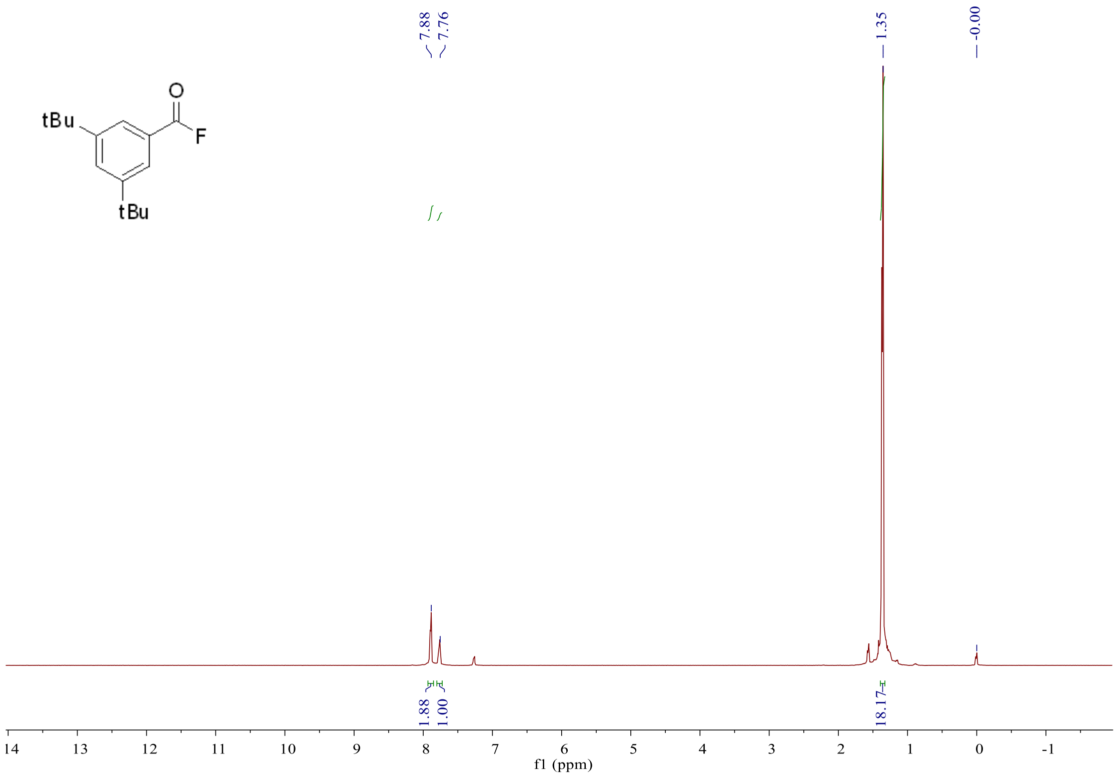

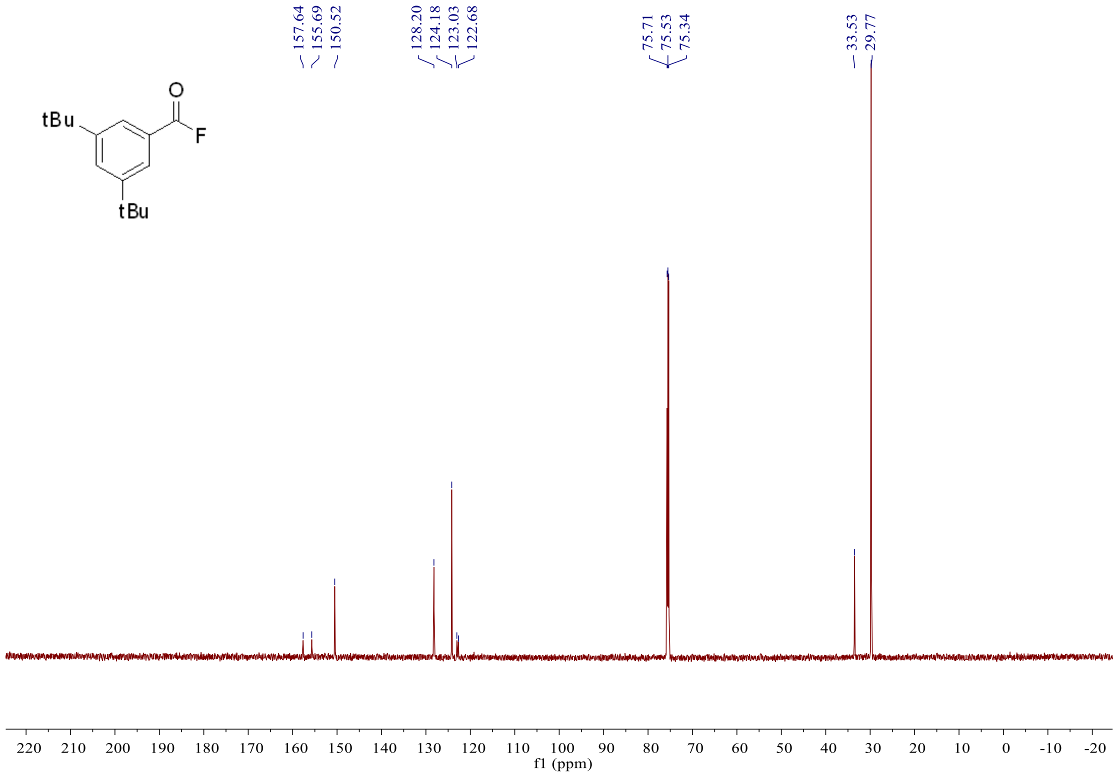

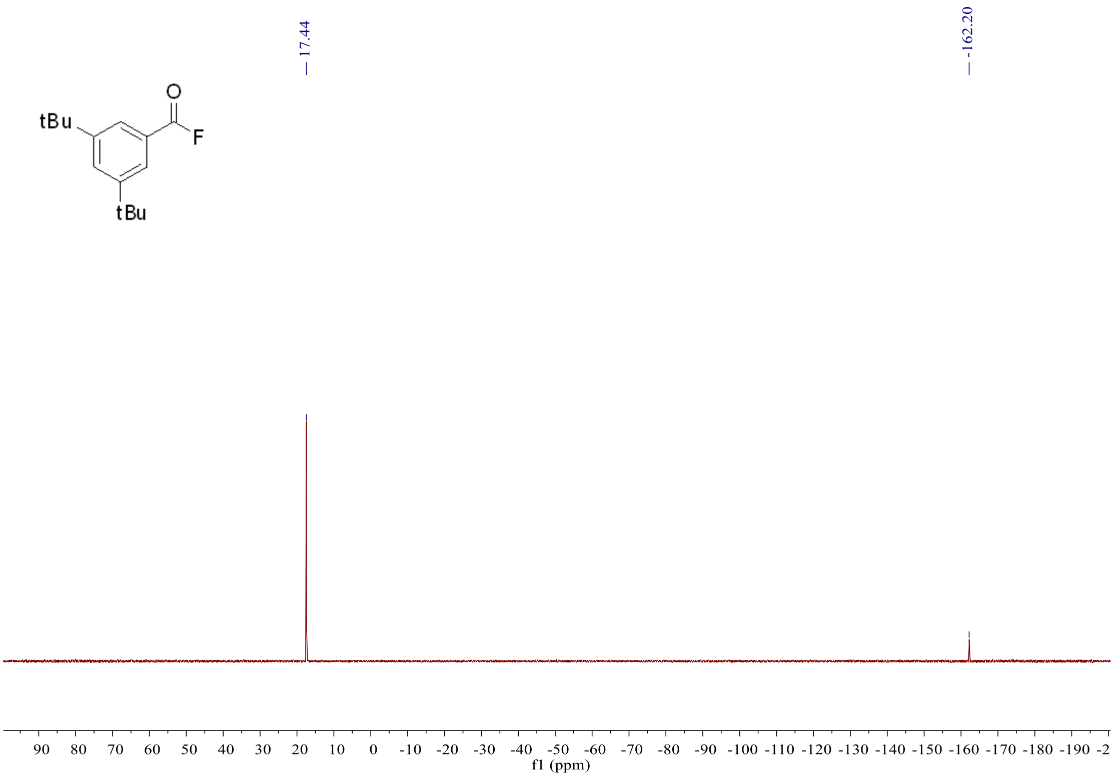

4-bromo-3-methylbenzoyl fluoride (1k).

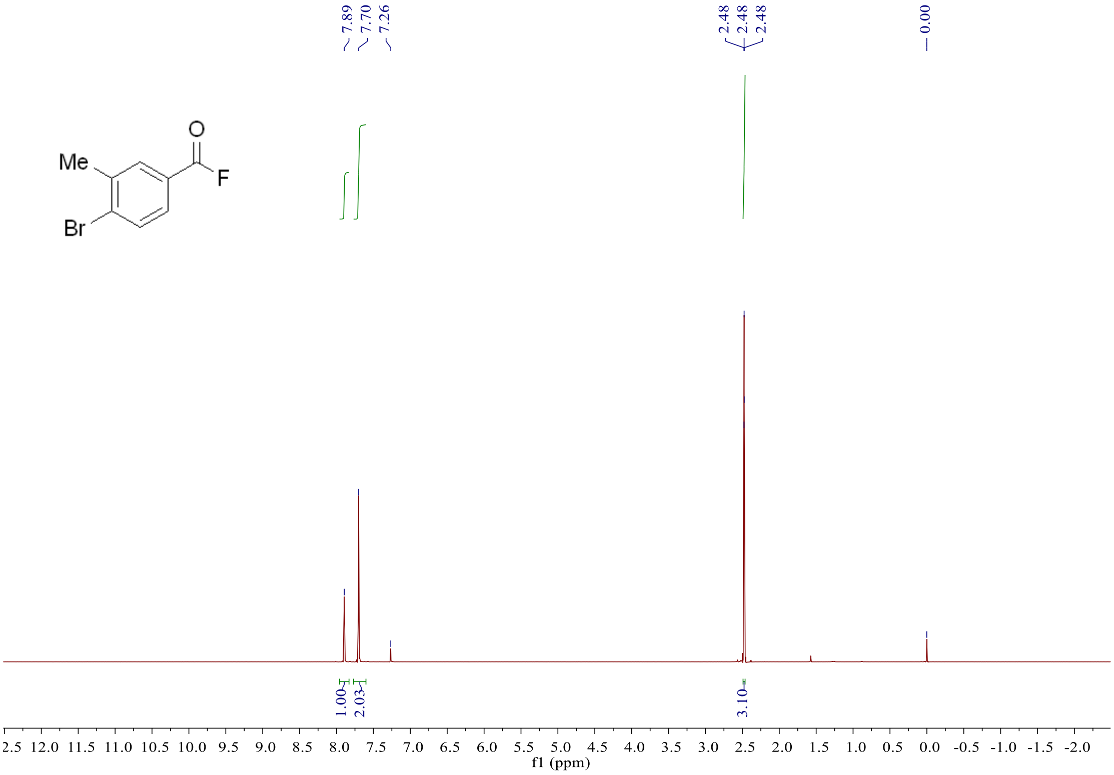

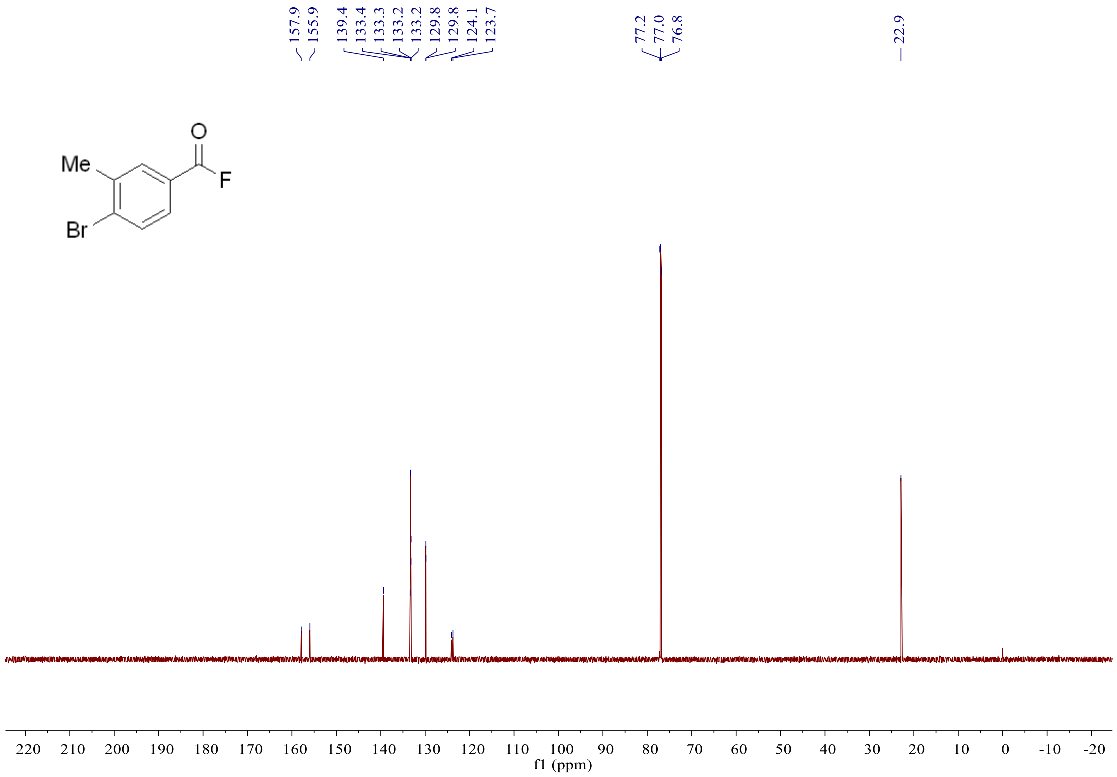
157.9
155.9
139.4
133.4
133.3
133.2
133.2
129.8
129.8
124.1
123.7
77.2
77.0
76.8
22.9
Me
Br
O
F
220 210 200 190 180 170 160 150 140 130 120 110 100 90 80 70 60 50 40 30 20 10 0 -10 -20
f1 (ppm)

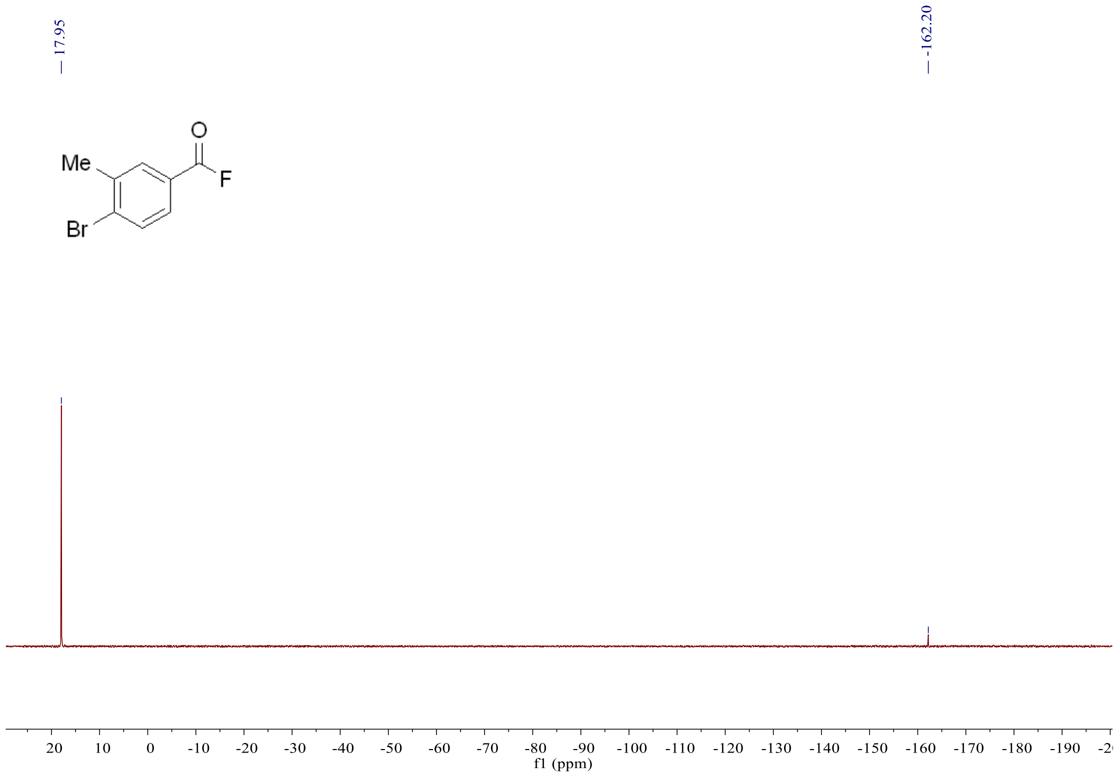
17.95
-162.20
Me
Br
O
F
20 10 0 -10 -20 -30 -40 -50 -60 -70 -80 -90 -100 -110 -120 -130 -140 -150 -160 -170 -180 -190
f1 (ppm)

3,4-diethoxybenzoyl fluoride (1l).

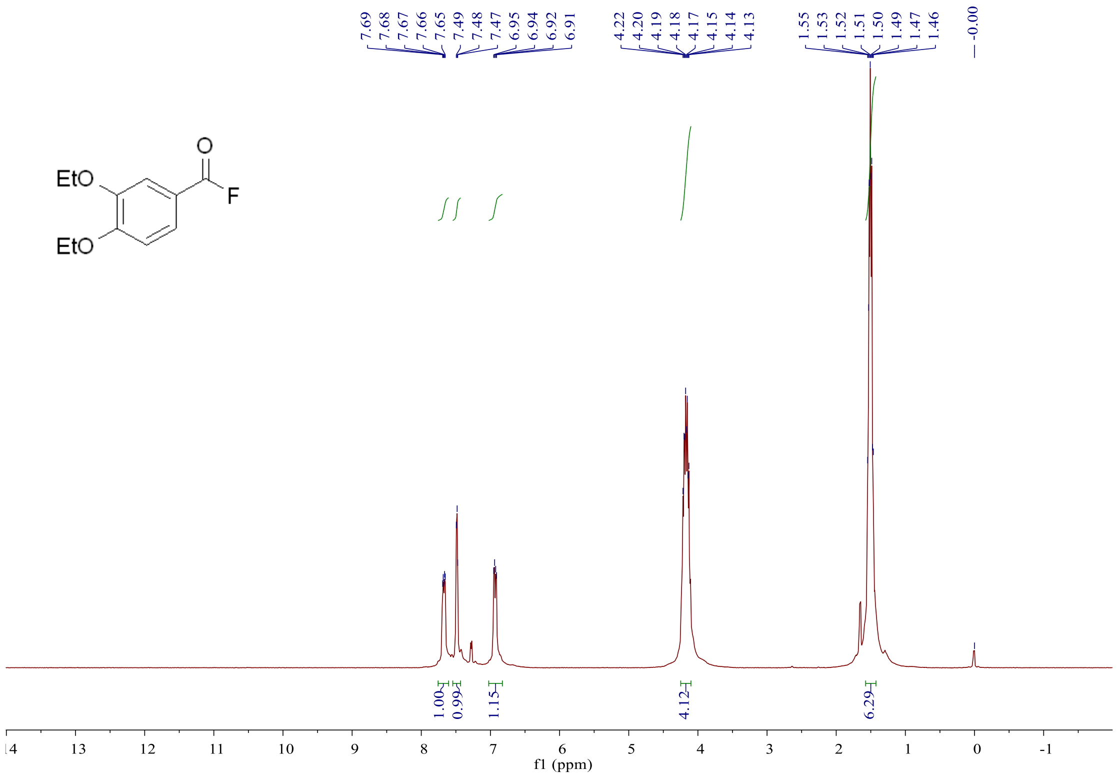

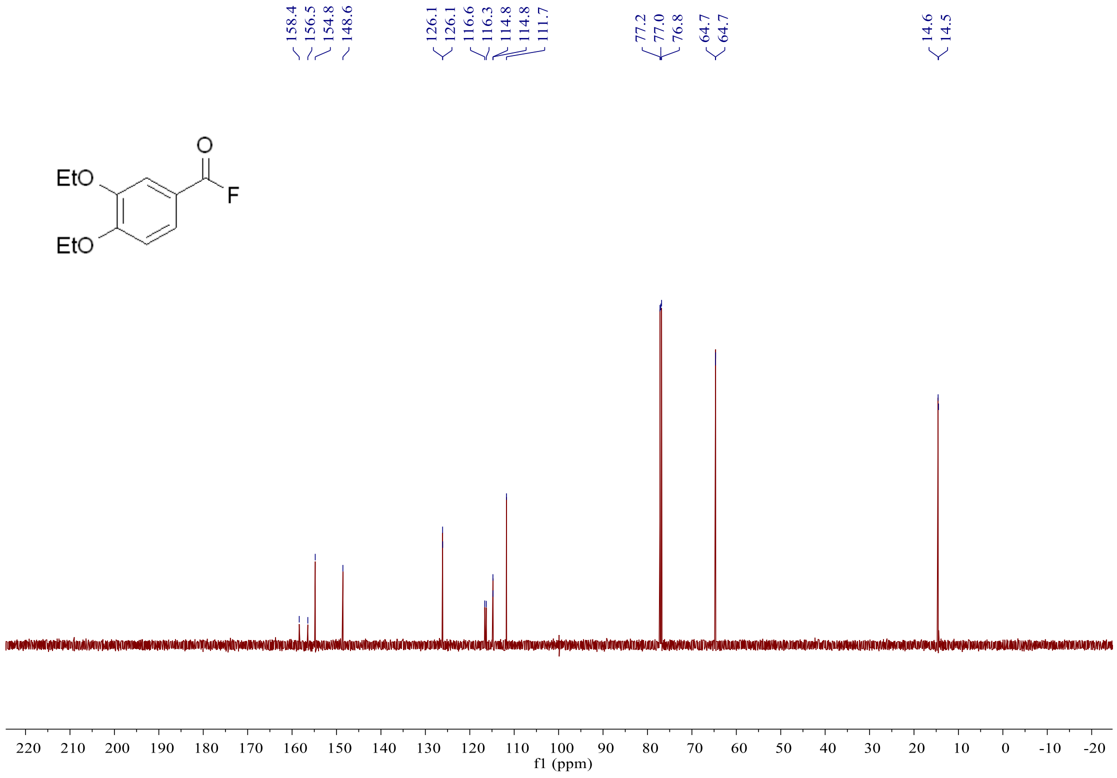

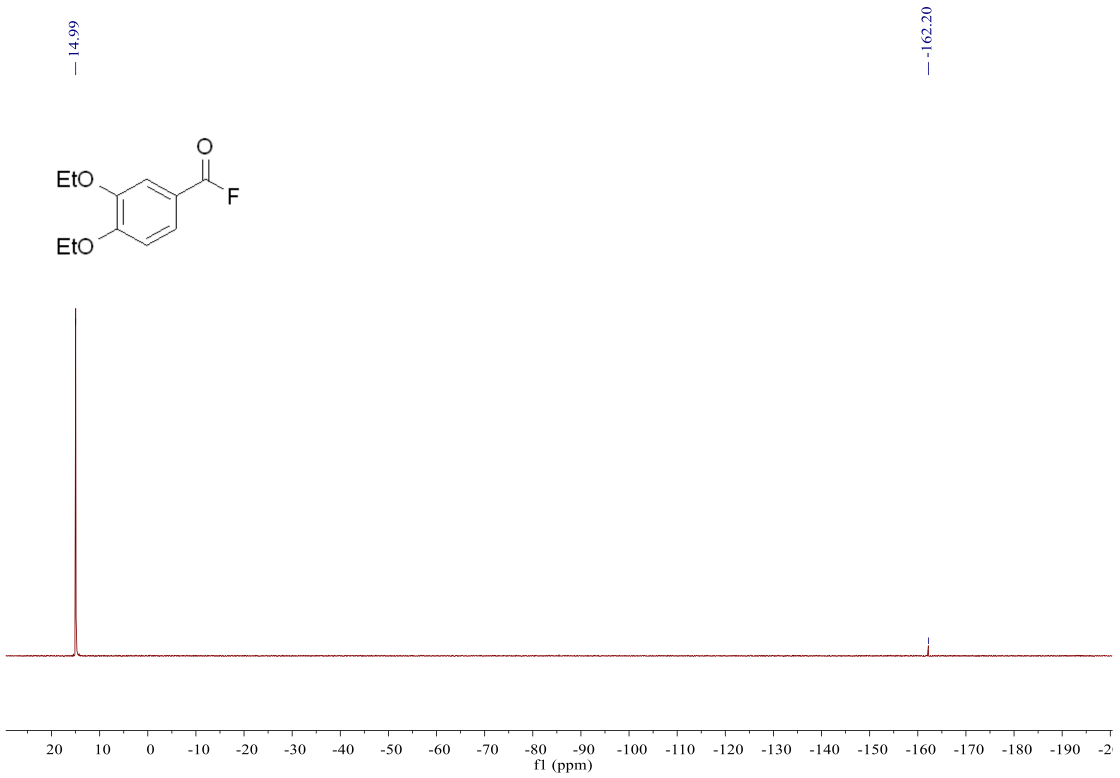
14.99
-162.20
EtO
EtO
O
F
20 10 0 -10 -20 -30 -40 -50 -60 -70 -80 -90 -100 -110 -120 -130 -140 -150 -160 -170 -180 -190
f1 (ppm)
